# Core gut microbes *Cloacibacterium* and *Aeromonas* associated with different gastropod species could be persistently transmitted across multiple generations

**DOI:** 10.1186/s40168-023-01700-0

**Published:** 2023-11-29

**Authors:** Datao Lin, Jinni Hong, Benjamin Sanogo, Shuling Du, Suoyu Xiang, Jerome Ho-Lam Hui, Tao Ding, Zhongdao Wu, Xi Sun

**Affiliations:** 1 Department of Parasitology, Key Laboratory of Tropical Disease Control (Ministry of Education), Zhongshan School of Medicine, Sun Yat-Sen University, Guangzhou, China; 2 Provincial Engineering Technology Research Center for Diseases-vectors Control and Chinese Atomic Energy Agency Center of Excellence on Nuclear Technology Applications for Insect Control, Sun Yat-Sen University, Guangzhou, China; 3 Department of Traditional Chinese Medicine, Guangdong Provincial People’s Hospital, Guangdong Academy of Medical Sciences, Southern Medical University, Guangzhou, China; 4 Laboratory of Parasitology, Institut National de Recherche en Sante Publique, Bamako, Mali; 5 State Key Laboratory of Agrobiotechnology, School of Life Science, The Chinese University of Hong Kong, Hong Kong, China

**Keywords:** Gastropod, Gut microbiome, Microbiome dynamics, Vertical transmission, Horizontal transmission, Developmental stage, *Biomphalaria*, Intermediate host, Vector, Snail-borne diseases

## Abstract

**Background:**

Studies on the gut microbiota of animals have largely focused on vertebrates. The transmission modes of commensal intestinal bacteria in mammals have been well studied. However, in gastropods, the relationship between gut microbiota and hosts is still poorly understood. To gain a better understanding of the composition of gut microbes and their transmission routes in gastropods, a large-scale and long-term experiment on the dynamics and transmission modes of gut microbiota was conducted on freshwater snails.

**Results:**

We analyzed 244 microbial samples from the digestive tracts of freshwater gastropods and identified Proteobacteria and Bacteroidetes as dominant gut microbes. *Aeromonas*,* Cloacibacterium*, and *Cetobacterium* were identified as core microbes in the guts, accounting for over 50% of the total sequences. Furthermore, both core bacteria *Aeromonas* and *Cloacibacterium*, were shared among 7 gastropod species and played an important role in determining the gut microbial community types of both wild and cultured gastropods. Analysis of the gut microbiota at the population level, including wild gastropods and their offspring, indicated that a proportion of gut microbes could be consistently vertically transmitted inheritance, while the majority of the gut microbes resulted from horizontal transmission. Comparing cultured snails to their wild counterparts, we observed an increasing trend in the proportion of shared microbes and a decreasing trend in the number of unique microbes among wild gastropods and their offspring reared in a cultured environment. Core gut microbes, *Aeromonas* and *Cloacibacterium*, remained persistent and dispersed from wild snails to their offspring across multiple generations. Interestingly, under cultured environments, the gut microbiota in wild gastropods could only be maintained for up to 2 generations before converging with that of cultured snails. The difference observed in gut bacterial metabolism functions was associated with this transition. Our study also demonstrated that the gut microbial compositions in gastropods are influenced by developmental stages and revealed the presence of *Aeromonas* and *Cloacibacterium* throughout the life cycle in gastropods. Based on the dynamics of core gut microbes, it may be possible to predict the health status of gastropods during their adaptation to new environments. Additionally, gut microbial metabolic functions were found to be associated with the adaptive evolution of gastropods from wild to cultured environments.

**Conclusions:**

Our findings provide novel insights into the dynamic processes of gut microbiota colonization in gastropod mollusks and unveil the modes of microbial transmission within their guts.

Video Abstract

**Supplementary Information:**

The online version contains supplementary material available at 10.1186/s40168-023-01700-0.

## Introduction

Virtually, all animals harbor a diverse gut microflora that greatly influences host health and diseases [1, 2]. Within the animal kingdom, the phylum Mollusca is the second largest, with gastropods being the most diverse class, comprising over 75,000 snail species worldwide. Gastropods, such as *Pomacea canaliculata* and *Biomphalaria straminea*, have gained global attention as invasive species originating from South America [3–5]. Some invasive freshwater gastropods not only serve as carriers for parasites but also pose a potential threat to agricultural products worldwide, thus jeopardizing human health and national security [6–8]. Concerns have recently been raised about the potential transmission risk of parasites such as *Schistosoma mansoni* and *Angiostrongylus cantonensis* by *B. straminea*, *Biomphalaria glabrata*, *Biomphalaria pfeifferi*, *P. canaliculata*, or *Physa acuta* [3, 5, 9–11]. Consequently, there is a call for alternative strategies to prevent such parasite transmission. The gut microbiota plays a crucial role in enhancing host immunity and nutrient digestion and regulating various metabolic pathways of the hosts. Therefore, understanding the involvement of gut bacteria in host health is essential to develop potential control strategies and gain a better understanding of the biological characteristics of gastropods. However, there is a limited amount of in-depth research regarding the “core” gut microbes of freshwater gastropods.

Previous studies have demonstrated that the majority of gut microorganisms in mammals are inherited vertically from their parents [12–14]. However, certain groups of gut microbiota, such as Bacilli and obligate aerobes, are predominantly acquired horizontally. In the case of plants, many microorganisms (bacteria and fungi) associated with the mother organism are transmitted vertically, for example, among *Glechoma hederacea* individuals [15]. While there have been studies focusing on describing the gut bacteria of gastropods in recent years [16–19], the transmission routes of the gut microbiota in gastropods have mostly been overlooked. By investigating the gut microbiota of gastropod species, we can gain a better understanding of the relationships between gastropod hosts and their commensal and/or symbiotic gut microorganisms.

Studies on adaptive evolution have mainly focused on the host genome or phenotypic diversity in animals [20–24], often disregarding the role of the gut microbiota, which also contributes to host functions and adaptations by providing essential resources during adaptive evolution. The convergent gut microbiota observed in African cichlid fish and mammalian species has been shown to contribute to host adaptation to diverse environments through the production of various compounds such as volatile fatty acids, highlighting the fundamental role of the microbiota in driving host adaptation processes [25, 26]. However, these studies have mostly examined the convergence of gut microbiota at a time point when the hosts have already fully adapted to the environment [25–27]. The dynamic process from initiation to convergence of host gut microbiota remains poorly understood. Therefore, a central ecological question remains: how have the long-term dynamic changes and convergence of the gut microbiota in gastropods occurred during host evolution?

In this study, our objective is to investigate key information about the “core” bacteria and transmission routes of the gut microbiota in gastropods. Specifically, we aim to answer the following questions:

i. What are the “core” bacteria present in the gut microbiota of both wild and cultured populations of freshwater gastropods?
ii. How does the gut microbiota of gastropods change across developmental stages?
iii. What are the transmission modes of the gut microbiota in gastropods? How are the “core” bacteria transmitted between habitats, and how do they persist over a generational time scale?
iv. Do the long-term dynamic changes in the gut microbiota follow the path of gastropod evolution? If so, can shifts in the gut microbiota serve as potential predictors of the time frame for gastropod adaptation to a new environment?

## Results

### Overview of collected and sequenced sample information

A total of 244 samples were collected from 7 wild or cultured freshwater gastropods: *Biomphalaria straminea* (*n* = 190), *Bellamya aeruginosa* (*n* = 14), *P. acuta* (*n* = 10), *Planorbarius corneus* (*n* = 12), *B. glabrata* (*n* = 7), *B. pfeifferi* (*n* = 8), and *P. canaliculata* (*n* = 3). The samples were investigated via the 16S rRNA gene (V3–V4 regions). Details about the number of samples sequenced, species, and generation information are shown in Additional file 1. After sequence quality control, an average of 48,670 (± 1101 SEM), 46,707 (± 1134), 46,651 (± 1137), 46,384 (± 1138), 45,330 (± 1124), and 42,153 (± 1085) sequences were classified into kingdom, phylum, class, order, family, and genus, respectively, per sample.

### Core Aeromonas and Cloacibacterium are common gut microbes and associated with different gastropod species and sample types

Over 50 genera of gut bacteria were identified from wild and cultured freshwater gastropods, with two dominant phyla, Proteobacteria and Bacteroidetes (Table S1; Figs. S1, S2). Among them, *Cloacibacterium*, *Aeromonas*, and *Cetobacterium* were consistently identified as the dominant gut microbes in both cultured and wild populations. The top 10 gut microbes from 7 wild-caught or cultured gastropod species are shown in Fig. S2.

**Fig. 1:**
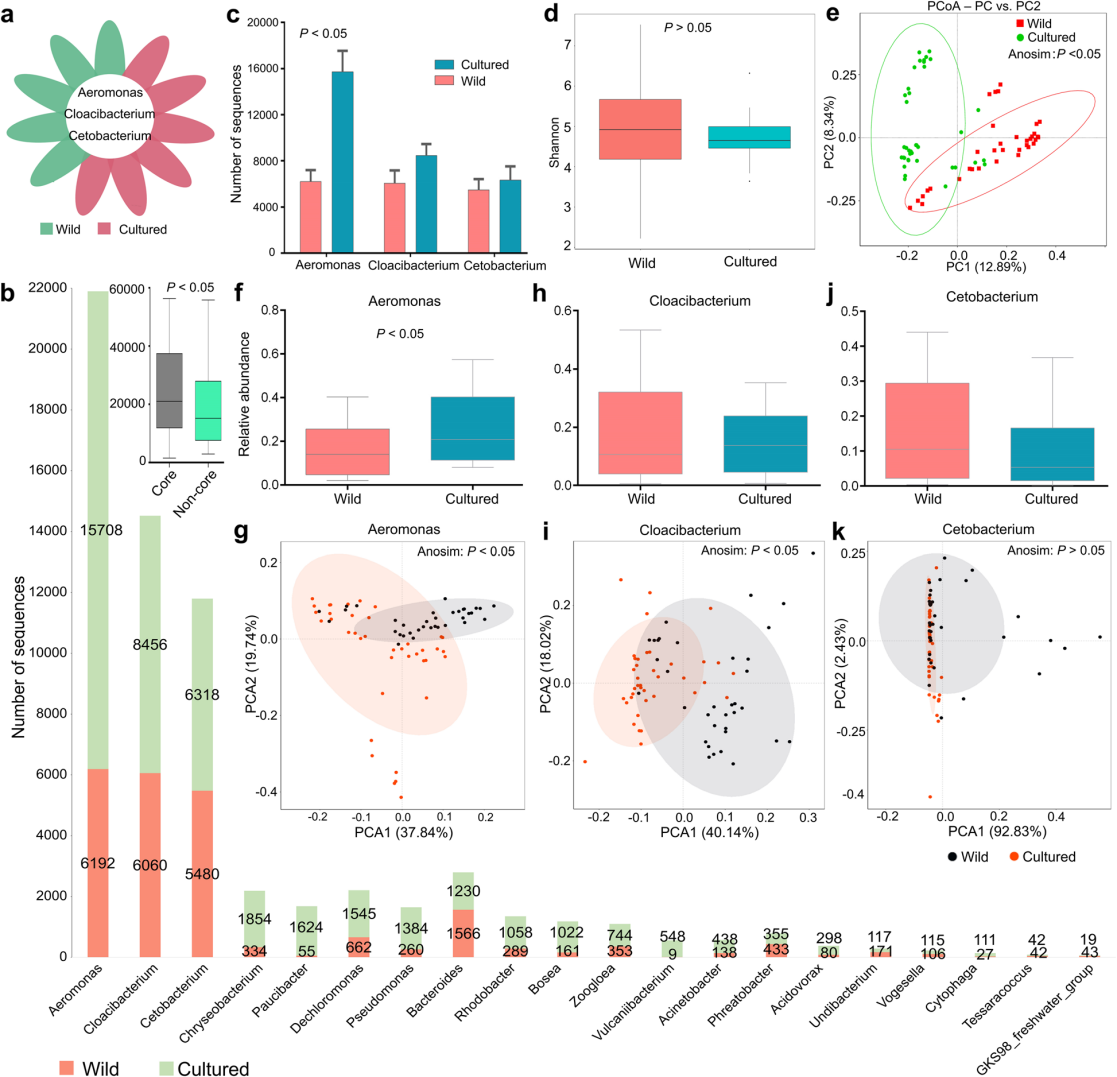
Comparison of the gut microbes in wild and cultured gastropod samples. **a** Venn diagram of the “core” microbes shared in wild-caught and cultured freshwater gastropods. At the genus level, 3 microbes that were shared among all snail species were defined as core microbes. **b** Rank abundance plot of the top 20 OTUs in wild and cultured gastropods. Core indicates sequences of core microbes. Noncore indicates sequences of noncore microbes but without unknown sequences. **c** Comparison of the OTU number of “core” microbes between wild and cultured samples. **d** Shannon diversity. **e** Distances shown in the PCoA plot are based on binary Jaccard. **f**–**g** Relative abundance and PCA are based on *Aeromonas*. **h**–**i** Relative abundance and PCA are based on *Cloacibacterium*. **j**–**k** Relative abundance and PCA are based on *Cetobacterium*. Cultured snails refer to the laboratory snail reared under laboratory conditions

To further understand the difference between wild and cultured populations, we examined the “core” microbes. This study showed that *Aeromonas*,* Cloacibacterium*, and *Cetobacterium* were the common dominant gut microbes across the different gastropod species (Fig. 1a; Figs. S1, S2). While *Aeromonas* belonged to the Proteobacteria phylum and *Cloacibacterium* belonged to Bacteroidetes, *Cetobacterium* belongs to the nondominant phylum Fusobacteria (Fig. S1). The median number of the top 20 operational taxonomic units (OTUs) belonging to a unique microbe in gastropods is shown in Fig. 1b. A genus-level analysis revealed that *Aeromonas*, *Cloacibacterium*, and *Cetobacterium* accounted for the majority of the sequences in the gut microbiota, indicating their dominance in both wild and cultured populations (Fig. 1b; Fig. S3a). The number of OTUs belonging to these three genera was generally higher in cultured snails compared to wild snails (Fig. 1c).

Comparing the gut microbial communities at the genus level, this study found that wild freshwater snails exhibited a similar level of microbial richness as cultured gastropods (Fig. 1d). The PCoA plot demonstrated distinct clustering of gut microbial community structures between wild and cultured snail populations (Fig. 1e). Several gut microbes, such as Proteobacteria, *Desulfovibrio*, and *Aeromonas*, were identified as biomarkers for distinguishing between wild and cultured snails using a linear discriminant analysis (LDA) and effect size (LEfSe) analysis (Fig. S3b). Furthermore, there were genus-level differences in gut microbial communities of wild and cultured populations (Fig. 1c, f–i), although no significant difference was observed based on the *Cetobacterium* data (Fig. 1j, k).

### The core microbes Aeromonas and Cloacibacterium can be transmitted across different habitats

**Fig. 2:**
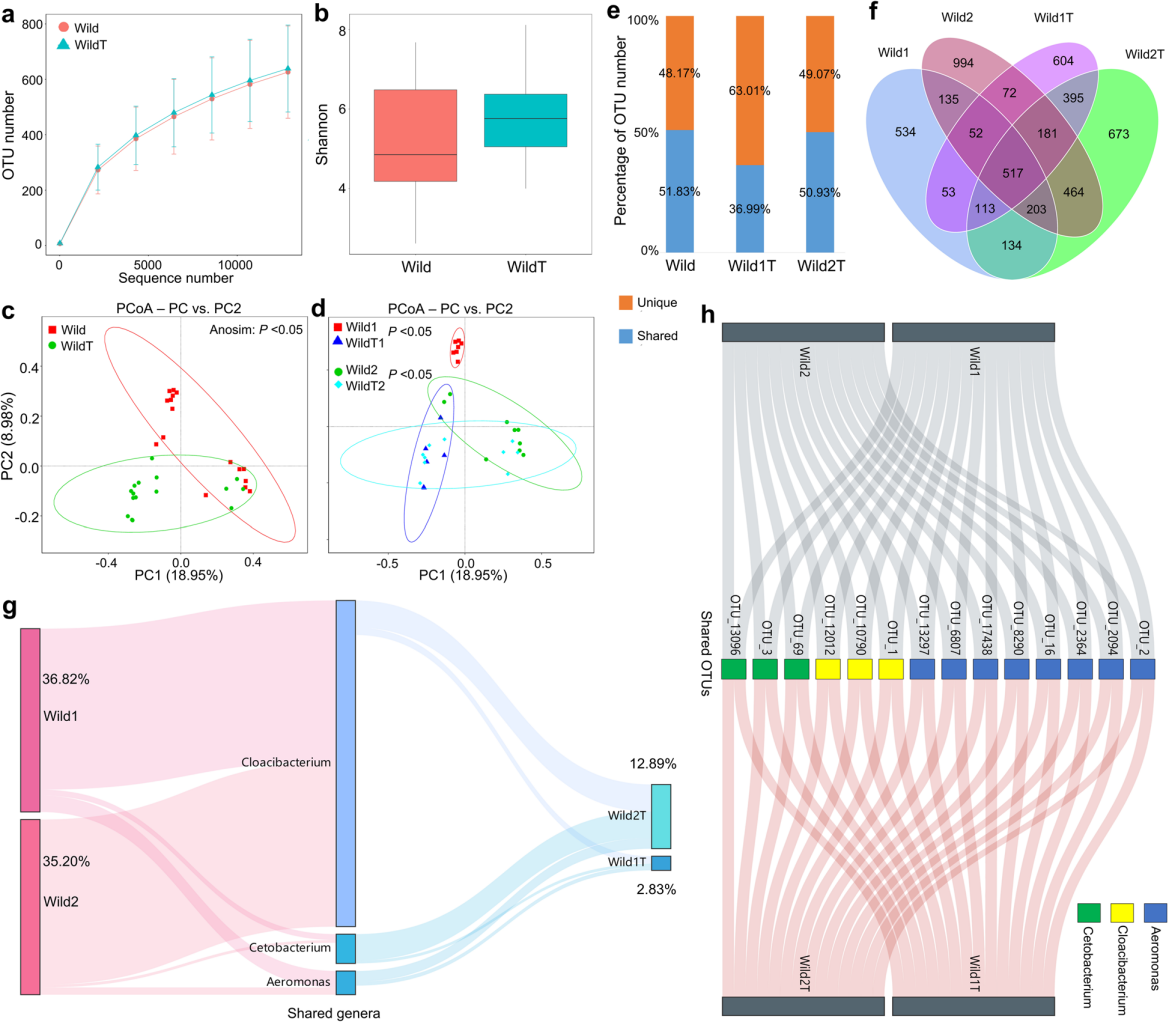
Horizontal transmission of the gut microbiota in gastropods. **a** Rarefaction analysis of observed OTUs for the gut microbiota of Wild and WildT populations. **b** Shannon index. **c**–**d** PCoA of the gut microbiota among populations. **e** Shared and unique OTUs between Wild and WildT populations, between Wild1 and Wild1T, and between Wild2 and Wild2T. **f** Venn diagram showing shared and unique OTUs among the populations. **g** Sankey diagram showing the core microbes among the populations. **h** Shared OTUs belonging to core microbes. Wild, wild-caught snails. WildT, WildT1, and WildT2, wild-caught snails being transferred to the laboratory. Wild1 and Wild2 represent sampling site 1 and site 2 in field studies

The gut microbiota composition between wild and cultured snails was found to differ, but they still shared core *Aeromonas* and *Cloacibacterium* microbes. However, changes in the gut microbiota of snails following transfer to new habitats are not well understood. In this study, we investigated the changes in the gut microbiota of wild *B. straminea* gastropods (Wild) after transfer to a laboratory environment (WildT) within 50 days and analyzed the horizontal transmission of gut bacteria in freshwater gastropods (Table S2). We then compared the shifts in gut microbiota diversities and community structures of wild *B. straminea* after breeding in the laboratory. No significant differences were found in OTU number (Fig. 2a) or alpha diversity (Fig. 2b) between the Wild and WildT groups. However, regardless of sampling locations (site 1 or site 2), WildT snails harbored a gut microbiota that was compositionally distinct compared to that in wild *B. straminea* snails (Fig. 2c, d; Table S3).

At the genus level, certain bacterial taxa, such as potential pathogenic *Pseudomonas* and *Flavobacterium*, were more prevalent in the WildT *B. straminea* snails compared to the wild snails (Fig. S4). However, the relative abundances of *Aeromonas* and *Cloacibacterium* sharply declined in the gut microbiota of WildT snails after they were transferred to the laboratory. We discovered that about 51.83% of the total OTUs present in the gut microbiota of wild *B. straminea* could be transmitted to the WildT snails (Fig. 2e). Interestingly, over 63% of the total OTUs observed in the Wild1T group could not be traced to wild snails (Fig. 2e). This suggests that the majority of gastropod gut microbiota undergo horizontal transmission, and a large portion of microbes originating from the environment or unknown sources may play a role in this transmission after snails are transferred to a new habitat. Additionally, we identified 517 core OTUs (10.1% of the total OTUs) in the gut microbiota originating from both wild-type and WildT snails using the Venn diagram (Fig. 2f). Some shared OTUs, such as OTU_1, OTU_2, and OTU_3 (belonging to the genera *Aeromonas*, *Cloacibacterium*, and *Cetobacterium*, respectively), were found among both wild-type and WildT snails (Fig. 2g, h).

### The core microbes Aeromonas and Cloacibacterium can be vertically transmitted across multiple generations

**Fig. 3:**
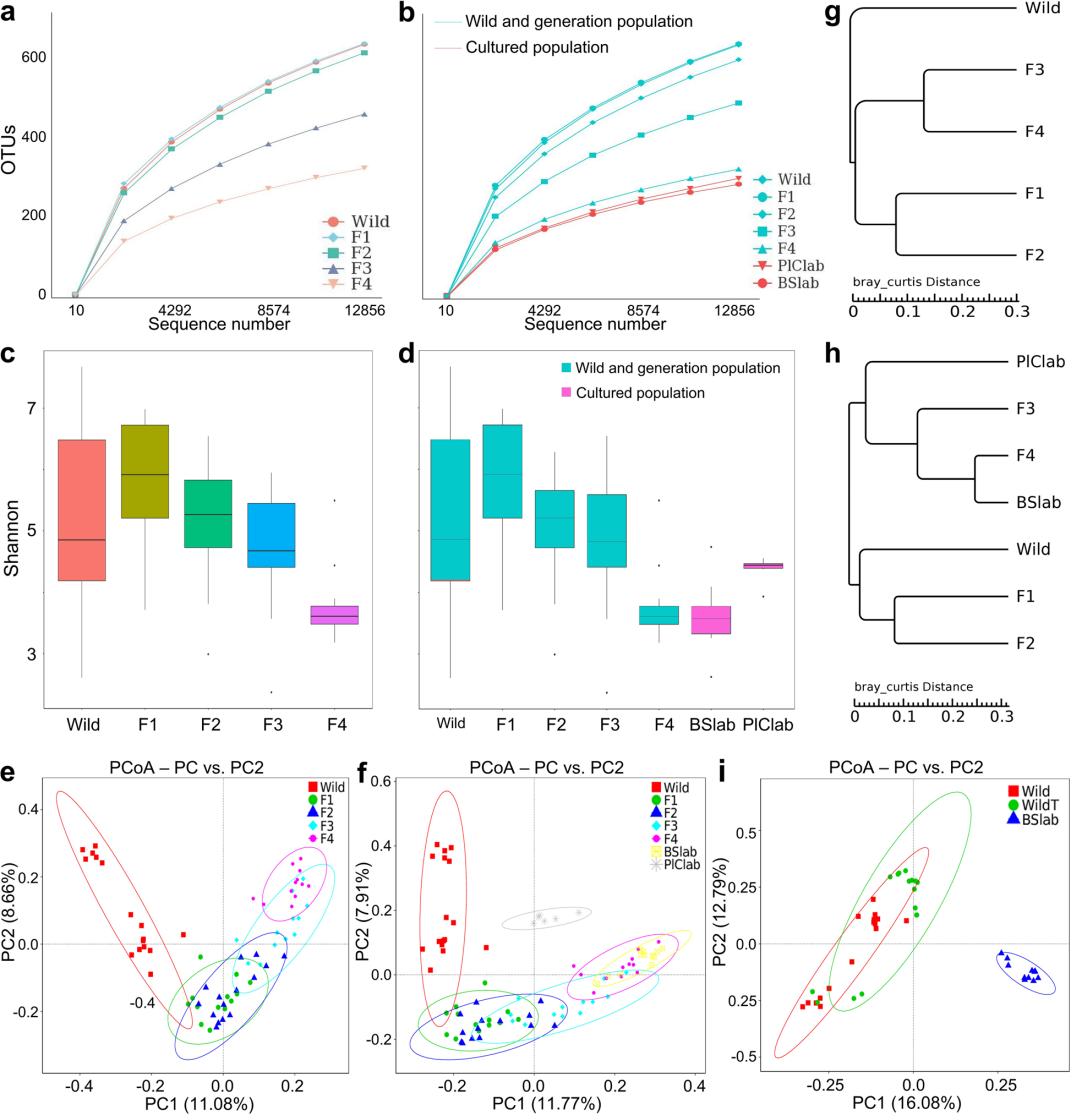
Overview of vertical shifts of gut microbes in gastropods over generations. **a**–**b** Rarefaction analysis of observed OTUs. **c**–**d** Shannon index. **e**–**f** PCoA was conducted based on the binary Jaccard dissimilarity among groups. **g**–**h** UPGMA analysis was conducted based on Bray–Curtis distance. **i** PCoA was conducted based on the binary Jaccard dissimilarity among groups. Cultured snails refer to the laboratory snails cultured under laboratory conditions

**Fig. 4:**
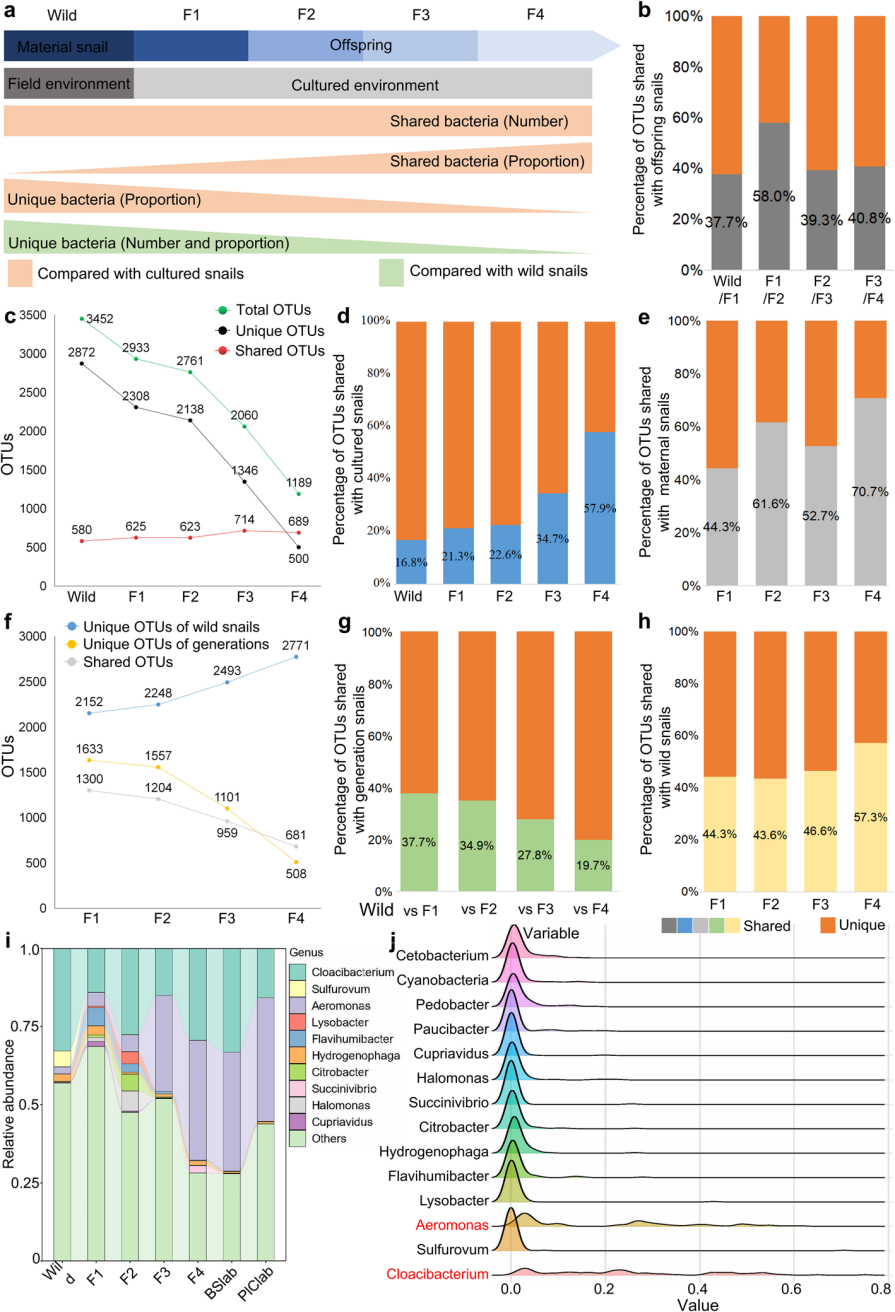
Overview of the vertical transmission of the gut microbiota among wild-caught and laboratory-bred individuals among gastropod lineages. **a** Overview of vertical shifts of gut microbes in gastropods. **b** Percentage of OTUs transmitted to offspring snails. For example, the left frame shows that 37.7% of total OTUs from wild snails can be transmitted to the F1 generation population. **c**–**d** The number and proportion of OTUs are shared or unique to sample types, compared with cultured BSlab snails. **e** Percentage of OTUs among populations that can be traced to maternal snails. **f**–**g** The number and proportion of OTUs are shared or unique to sample types, compared with wild-caught snails. **h** Percentage of OTUs shared with wild snails. **i** Sankey diagram showing the core microbes among populations. **j** Ridgeline chart showing the distribution shifts among the top gut microbes in gastropods over generations. Cultured snails refer to the laboratory snails cultured under laboratory conditions

In this study, we conducted experiments to investigate the vertical transmission of the gut microbiota in gastropods. We collected wild gastropods and bred them in the laboratory, producing F1, F2, F3, and F4 generations. Additionally, we aimed to explore the potential relationship between the gut microbiota and host adaptation (Figs. 3 and 4; Table S4).

We quantified the abundance, alpha diversity, and community structure of the gut microbiomes, revealing dynamic variances in the gut microbiota through vertical transmission over 4 generations. No significant difference was observed in the alpha diversities of the gut microbiota between the F1 and F2 generations and the wild snails (maternal snails) (*P* > 0.05) (Fig. 3a–d). However, significant shifts in bacterial community structures were observed in the offspring (Fig. 3e, f and Table S5).

Notably, the alpha diversities and community structures of the gut microbiota changed significantly in the F3 and F4 generations, gradually resembling those observed in cultured populations (Fig. 3b, d, e, f). Interestingly, the wild, F1, F2, and F3 populations exhibited significantly different gut bacterial compositions (*P* < 0.05), while the F4 and the combination of the F3 and F4 generations showed a gut microbiota similar to that of the laboratory gastropods (*P* > 0.05) (Fig. 3a–e; Table S5). Furthermore, UPGMA cluster analysis demonstrated convergent gut microbial structures in the F3, F4, laboratory *B. straminea* (BSlab), and laboratory *P. corneus* (PlClab) populations, distinct from the clusters of the F1, F2, and wild gastropod clusters (Fig. 3g, h). In addition, there was a significantly different gut microbiota composition in WildT snails compared to the wild and BSlab snails (Fig. 3i; Tables S3 and S5).

We then analyzed the shifts of microorganisms from wild to cultured gastropod populations and investigated the differences in gut bacterial composition between mothers and offspring. For this part, we defined gut microbes in wild gastropods as the “wild” microbiome, while features in the gut of cultured snails were defined as “cultured” microbes. We observed an increasing trend of shared bacteria in snails reared in a cultured environment and a decreasing trend in the number of unique bacteria (Fig. 4a). Except for the F1 generation (the maternal of F2), less than 50% of the total OTUs harbored in maternal gastropods are vertically inherited by the offspring (Fig. 4b, Fig. S5), indicating that only a proportion of the gut microbiota in gastropods is vertically transmitted from mother to offspring. Notably, the number of total and unique gut bacteria in mothers and offspring declined sharply, while there was a small change in the shared microbes (Fig. 4c). Furthermore, the proportion of shared OTUs in the gut microbiota between mothers and offspring became increasingly similar to that of cultured snails, while the percentage of unique OTUs gradually decreased (Fig. 4d). Over successive generations, only 44.3 to 70.7% of OTUs harbored by offspring could be traced back to mother snails, with an increasing trend (Fig. 4e).

To further evaluate the transmission routes of gut microorganisms in gastropods, we compared the dynamic shifts of gut bacterial composition in snails reared in laboratory environments with those of wild-caught populations. The number of shared and unique OTUs housed in the gut of offspring snails was sharply reduced compared to that of wild gastropods (Fig. 4f). Conversely, the unique microbes increased in wild snails compared to their offspring. However, the percentage of gut microbes in offspring decreased gradually, which differed from the proportion of shared OTUs in descendants (Fig. 4g, h). Finally, we found that the distribution of the gut microbes *Aeromonas* and *Cloacibacterium* was more persistent and dispersed from wild snails to their offspring, while other microbes, such as *Cetobacterium*, only appeared at certain generations with a high relative abundance but were not persistent (Fig. 4i, j).

### The evolution and shifts of gut microbiota through vertical transmission over multiple generations are associated with distinct bacterial signatures

**Fig. 5:**
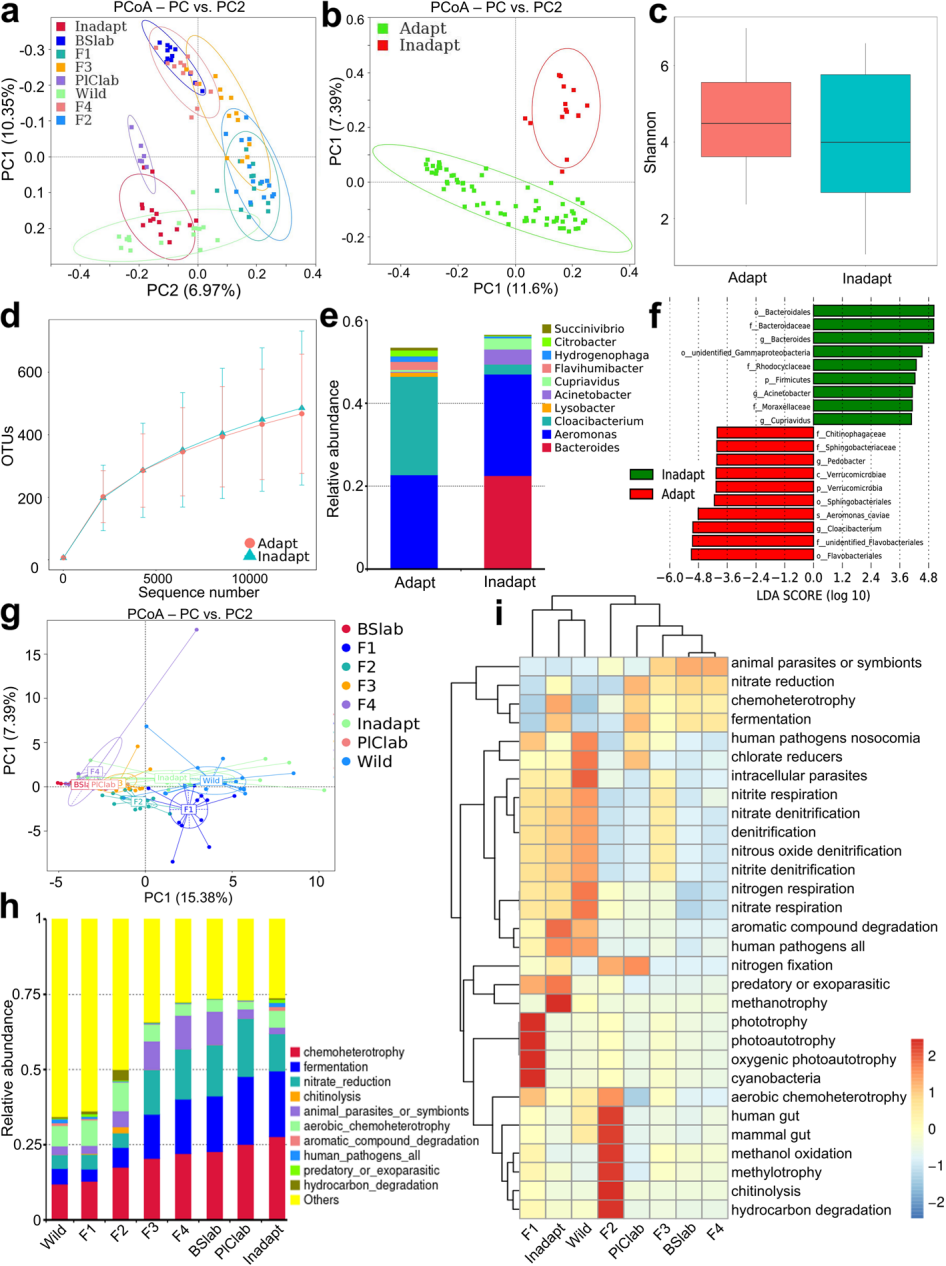
Shifts of gut microbiota are associated with metabolism over generations in gastropods. **a**–**b** PCoA on gut microbiota. **c** Shannon index of gut microbiota. **d** OTUs analysis of gut microbiota. **e** Gut microbiota showing at the genus level. **f** Differential gut bacterial taxa analyzed by LEfSe analysis with LDA score > 4 between groups. **g** PCoA on metabolism composition of gut microbiota among groups. **h** Relative abundance at metabolism levels in gut microbiota among groups is observed using FAPROTAX. **i** A heatmap of the relationship between metabolic functions and evolutionary process of gut microbiota enriched in gastropod populations. Cultured snails refer to the laboratory snails cultured under laboratory conditions

While some offspring of wild snails were able to successfully adapt to new habitats, others were unable to adapt to the cultured environment and became sick, eventually dying [28]. We found that nonadaptable snails harbored compositionally distinct gut microbiota compared to adaptable offspring and cultured snails (Fig. 5a, b; Table S6). However, the Shannon diversity value of gut microbiota in nonadaptable snails was similar to that of the adaptable group (Fig. 5c, d). Additionally, we observed significant variations in the abundance of certain bacterial taxa in nonadaptable *B. straminea*, such as *Aeromonas*,* Cloacibacterium*, *Bacteroides*, and *Acinetobacter* (Fig. 5e, f).

Nevertheless, it remains unclear how the gut microbiota of gastropods undergoes changes from the wild to cultured environments. Therefore, we further examined the potential metabolic functions of the gut microbiota in wild and cultured populations using FAPROTAX [29]. Our findings showed that after being moved from the wild into the laboratory, OTU functions in cultured gastropods converged and became enriched over generations, leading to their metabolic compositions becoming similar to those of cultured snail gut microbiota (Fig. 5g, h). Nonadaptable populations exhibited a more diverse microbial flora with lower diverse metabolic functions than adaptative snails. The microbial metabolic composition of the F2, F3, and F4 generations, as well as cultured snails, clustered under the same branch, which showed a higher metabolic functional abundance of animal microflora including possible parasites or symbionts, nitrate reducers, chemoheterotrophs, and fermenters (Fig. 5i). The F1 generation, as well as wild and nonadaptable gastropods, harbored similar microbial metabolic function collections. Despite the fact that nonadaptable snails harbored a similar array of dominant taxonomic microbial metabolites compared to cultured hosts (Fig. 5h), the relative abundance of other gut microbial functions, such as human pathogens, nosocomial organisms, and chlorate reducers, was similar to that of wild samples (Fig. 5i). Furthermore, we found that the balance of microbial interactions of core microbes in nonadaptable snails was disrupted, with the highest ratio of positive/negative edges (15.7) in their gut microbiota (Table S7).

### The core microbes Aeromonas and Cloacibacterium are transmitted across different developmental stages

We utilized the gastropod *B. straminea* as a model to conduct a longitudinal investigation of gut microbiomes across different life stages, collecting a total of 58 samples from 0-day-old (egg) to 450-day-old individuals. Life stages of *B. straminea* were defined as egg, youth, adult, and older groups based on age and reproductive capacity. Details of collected samples from important life stages are presented in Table S8.

**Fig. 6:**
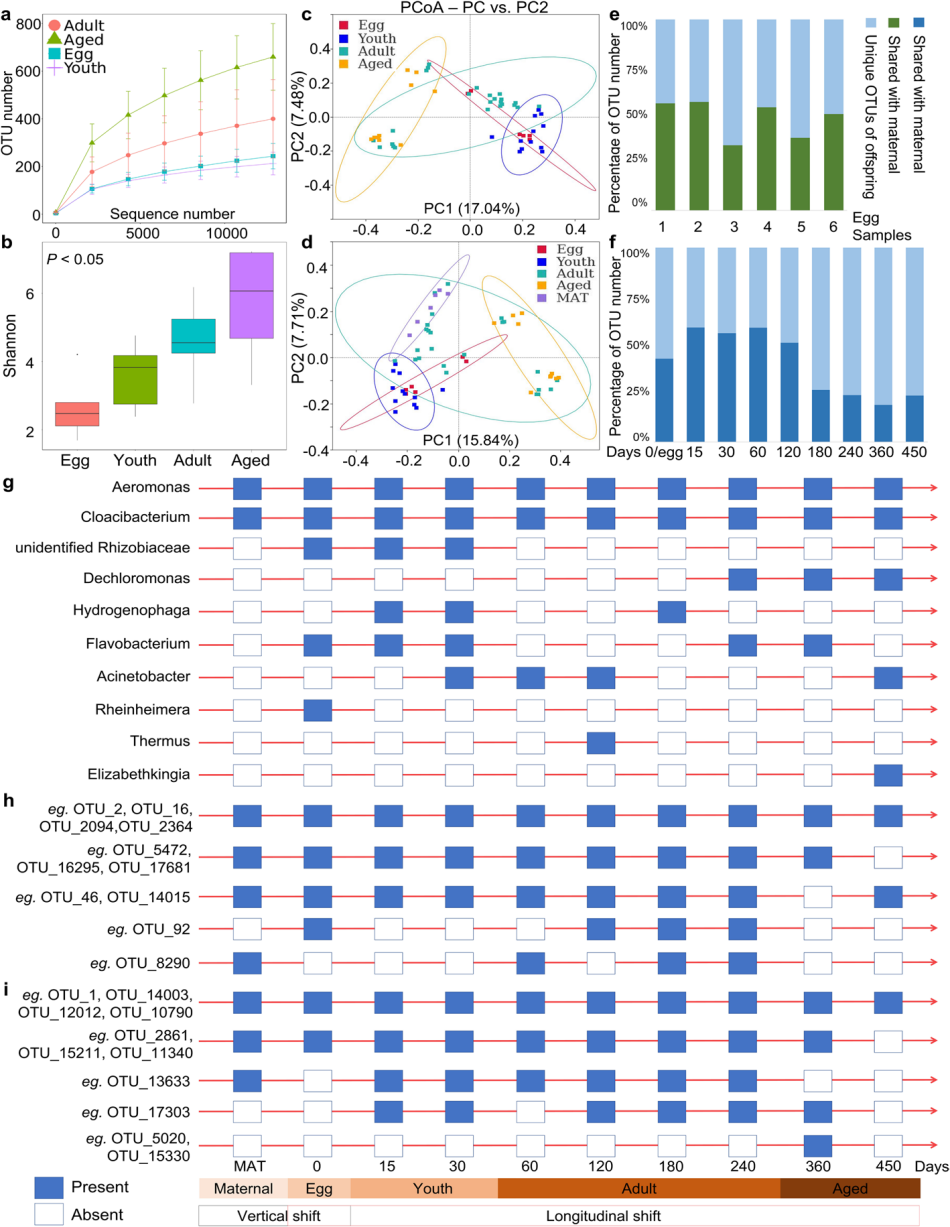
Overview of the longitudinal shifts of gut microbes across developmental stages in gastropods. **a** Rarefaction analysis of observed OTUs. **b** Shannon index. **c**–**d** PCoA based on the binary Jaccard dissimilarity among groups. **e**–**f** Percentage of OTUs that were unique or shared between snail population. **g** Longitudinal investigation patterns of gut microbes in gastropods with > 1% relative abundance. **h** OTUs belong to *Aeromonas*. **i** OTUs belong to *Cloacibacterium*. The blue and white frames represent the presence and absence of microbes during developmental stages or age points, respectively. Cultured snails refer to the laboratory snails cultured under laboratory conditions

Our analysis revealed an overall increasing trend of bacterial diversity from the egg stage to the older stage (Fig. 6a, b), as well as from 0 days old to 450 days old (Fig. S6). The gut microbiota from older snails exhibited the highest diversity, followed by the adult stage and youth stage, while the egg stage had the lowest richness (*P* < 0.05). The PCoA plot revealed a significant shift in community membership and structure from egg samples to the older stage, with older snails displaying a more diffuse distribution of gut bacterial communities (Fig. 6c, d; Table S9).

To examine core microbes across life stages, we analyzed the relative abundances of the predominant taxa. Proteobacteria was identified as the most abundant phylum in the microbiome across developmental stages, followed by Bacteroidetes, which together accounted for over 80% of the total sequences belonging to these two phyla (Fig. S7a). The prevalent microbial families and genera within *B. straminea* microbiota communities are presented (Fig. S7b, c). Notably, the genera *Aeromonas* and *Cloacibacterium* were consistently distributed across important developmental stages. Interestingly, the genus *unidentified *Rhizobiaceae dominated specifically in the egg stage, with a relative abundance greater than 60% compared to other stages. In addition, various gut microbial markers exhibited variations in different developmental stages (LDA score > 4; Fig. S8). For instance, among the top 20 microbes in the youth stage, the genera *Aeromonas*, *Cloacibacterium*, *Dechloromonas*, *Hydrogenophaga*, *Phreatobacter*, and *Pedobacter* were identified as stage dependent.

We further investigated the distribution of microbes during different developmental stages in gastropods and examined the longitudinal and life cycle shifts of core gut microbes. Additionally, we compared the composition of the gut microbiota between maternal snails and their offspring to gain a more comprehensive understanding. In our analysis, we found that, on average, 110 shared OTUs were identified in the egg stage when compared to maternal snails (Fig. S9a). Furthermore, we observed 352 shared OTUs between maternal snails and their offspring across life stages (Fig. S9b). The proportion of shared OTUs derived from maternal samples was approximately 48.2% in eggs and 40.16% in different-age snails (Fig. 6e, f). Interestingly, the proportion of shared OTUs in older snails was lower than that in younger ones (Fig. 6f).

We then investigated what types of gut microbes were consistently transmitted across generations and life cycles. The presence (with a relative abundance greater than 1%) and absence of gut microbiota are shown (Fig. 6g). The genera *Aeromonas* and *Cloacibacterium* were present in both maternal and egg samples and consistently appeared at all stages of the gastropod life cycle. This indicates that these two genera could be vertically and longitudinally transmitted during the gastropod life cycle. However, other microbes, such as unidentified Rhizobiaceae and *Rheinheimera*, only appeared at certain stages, such as the egg stage, and were absent in subsequent stages. We referred to these microbes as stage-associated organisms. Additionally, certain microbes, such as *Dechloromonas*, *Thermus*, and *Elizabethkingia*, appeared only at specific stages, further supporting their classification as stage-associated organisms.

By analyzing the 114 shared OTUs depicted in the Venn diagram (Fig. S9c), we conducted a more detailed examination of the order and timing appearance across developmental stages for the shared OTUs belonging to the genera *Aeromonas* and *Cloacibacterium* (Fig. 6h, i). For example, OTU_2, 16, 2094, and 2364 clustered under the genus *Aeromonas* and were consistently observed across generations and life cycles. In contrast, other OTUs, such as OTU_46 and 8290, appeared only at specific stages or during different ages.

### A predictive model for gastropod adaptation utilizing gut microbiota signatures

We observed a convergence of the gut microbiota of cultured snails (Mollusca:Gastropoda), with increasing similarity in alpha diversities (Fig. S10a) and convergent bacterial community structures (Fig. S10b). To explore the relationship between snail species, we constructed a nearest neighbor tree using *CO1* gene sequences of snail species collected from South China or referenced on NCBI (Fig. S10c). We identified thirty bacterial genera as potential predictors for distinguishing nonadaptable *B. straminea* from locally cultured snails using a random forest model (Fig. S10d–g). Among these, specific gut bacterial biomarkers such as OTU_2094 (genus: *Aeromonas*) were considered independent variables. In general, our findings may suggest that changes in the gut bacterial signatures across generations are associated with the snail’s adaptation to the new environment.

## Discussion

The diversity and community of the gut microbiota are extremely important for host development and health [12, 30]. Currently, our knowledge about gut microbiota transmission mainly derives from studies on mammals [14, 31]. Gastropoda comprises the largest number of species among mollusk classes. In recent years, scientists have focused on the bacterial diversity and microbial community composition in gastropods [16, 17, 32]. However, our understanding of the comprehensive role of the gastropod gut microbiota remains insufficient.

Our study showed that “core” *Aeromonas* and *Cloacibacterium* are consistently associated with various gastropod species and sample types. For example, the gastropod *B. straminea* successfully invaded China and colonized the Hong Kong area and Guangdong Province. *B. straminea*,* B. glabrata*, and *B. pfeifferi* are important organisms for studying interactions between the schistosome parasite *S. mansoni* and its intermediate hosts, which are distributed in both wild and cultured environments [33–38]. Therefore, understanding the role of the gut microbiota in both wild and cultured populations of the host is essential. Our study identified highly diverse gut microbial communities in both laboratory-bred and wild-collected gastropods, similar to the results of previous studies on arthropods [39] and mammals [14]. These findings further emphasize the importance of considering the role of the gut microbiota when conducting experiments associated with wild and cultured gastropods.

In addition, the gastropod gut microbiota in both wild and cultured populations was dominated by Proteobacteria and Bacteroidetes, a result that differs from the dominant taxa reported in mosquitoes [40, 41], fishes [42], pigs [43], and mice [14, 44]. Core microbes play important roles in the growth performance, production, and health during the host’s development [43]. Previous studies have also shown that the genera *Aeromonas* and *Cloacibacterium* were primarily abundant members of the gut microbiota in the gastropods such as *Theodoxus fluviatilis* [17] and *P. canaliculata* [45]. In herbivorous and nonherbivorous freshwater snails such as *Planorbella trivolvis*, *Aeromonas* and *Cloacibacterium* were the most abundant gut microbes [46], which is consistent with our findings. However, further research is necessary to fully understand the role of the core microbes in the gut microbiota of gastropods.

Our study is the first to reveal the modes of transmission of the gut microbiota in gastropods. The dynamics of gut microbiomes influence host health and adaptation. Transmission modes of gut bacteria in mammals, such as humans [30, 47, 48] and mice [2, 14], have been extensively studied, but very few studies have been conducted on the gastropod gut microbiota. In mammals, the majority of the gut microbiota is vertically inherited, but a minority of the gut microbiota is horizontally transmitted. Interestingly, our study revealed that the majority of the gut microbiota can be horizontally transmitted in gastropods. Previous studies have shown that 30 to 70% of total gut microbes in offspring (mammals) could be traced to the mother [48–52], and that the majority of the murine gut microbiota was vertically inherited [14]. Additionally, 15% of gut viral communities in offspring were acquired from their maternal gut microbiota [48]. These studies revealed that certain gut microbiomes observed in offspring could be vertically transmitted from their mother in mammals. In this study, we found that the wild gut microbiota in gastropods could be maintained for only 2 generations in a nonsterile laboratory environment before gradually converging to the gut microbiota of cultured species in the F3 or F4 generations. These findings were different from the results of previous studies, which showed that the gut microbiota of wild mice could maintain the wild microbiota for 4 or over than 10 generations in a specific pathogen-free (SPF) environment [2, 14]. We believe that environmental factors may be an important factor inducing these differences because environmental factors dominate host genetics in shaping the gut microbiota [53]. Most gastropod gut microbiota may be transmitted horizontally, implying that the gastropod gut microbiota may be more easily influenced by environmental factors than by host genotype. In total, our findings showed that certain gut microbiota, such as *Aeromonas* and *Cloacibacterium*, can be persistently vertically transmitted, while the majority of the gut microbiota is not vertically transmitted in a non-sterile environment.

Convergent evolution of microbiota has been observed in both vertebrates (such as fishes [25] and mammals [26, 54, 55]) and invertebrates (such as insects [56, 57]). Hosts can benefit from volatile short-chain fatty acids (SCFAs) produced by convergent microbiota to adapt to environmental stress [25, 26]. Our study suggests that metabolic functions may play a role in driving the convergent evolution of gastropod gut microbiota, based on differences in gut microbiota between wild and laboratory snails. We found that the environment may be the primary driver of convergence in the gut microbiome across gastropod genotypes, rather than host genetics [53]. However, further research is needed to understand how microbiota shifts are shaped by environmental factors. Our study implied that the convergent evolution of gut microbes contributed to the adaptative evolution of gastropods, and that the convergent evolution of the gut microbiota among gastropod species may be determined by metabolic functions such as chemoheterotrophy, nitrate reduction, and fermentation. Overall, the convergence in the gut microbiota indicates a potential selective pressure leading to similar microbial composition and community structures among cultured snails.

Coevolution and the hologenome are important concepts for understanding the mechanisms underlying host-microbiome interactions [58, 59]. These concepts emphasize the intimate relationship between the gut microbiota and its host, as well as the influence of host genetics on the gut microbiota composition and the ability of the gut microbiota to enhance host defense against pathogens. Rapid shifts in the gut microbiota, distinct from the gut microbiota of wild animals, were identified during human evolution [58, 60], suggesting that the link between gut microbiota and host adaptability may be associated with speciation [59]. Previous studies have demonstrated that host-microbiome interactions could affect both health and fitness [58], and the gut microbiota between healthy and unhealthy hosts is significantly distinct. The transmission modes of gut microbiota occurred with host evolution [14, 58]. Therefore, it is hypothesized that the transmission modes of gut microbiota, including vertical and horizontal transmission, may reflect the current evolutionary status of the host. The transmission mode of the gut microbiota may be associated with host adaptation. Our study revealed that most of the gut microbiota in gastropods can be horizontally transmitted, which is consistent with previous research [14]. Our data also showed that in laboratory snails, the abundance of potential pathogenic microorganisms became higher than that of wild-captured snails*.* Moreover, the survival rate of laboratory-bred wild snails sharply decreased, showing a positive association with the level of gut microbiota via horizontal transmission. An increase in potential pathogenic microbes may lead to higher mortality in the wild populations after being transferred to a new habitat. In addition, we revealed a trend in which a majority of “wild” gut microbes were removed, with the gut microbiota eventually becoming similar to the “cultured” gut bacteria after 3 to 4 generations. Finally, the “reserved” (vertically transmitted) gut microbiota in the offspring of wild snails showed a higher relative abundance of the microbes *Aeromonas* and *Cloacibacterium*, similar to those found in cultured snails. Host health is associated with transmission modes of gut microbiota in gastropods, especially vertical transmission. In short, vertical transmission, not horizontal transmission, may drive the convergent evolution of the gut microbiota to promote host health.

We have identified core gut microbes in snail guts that are shared across different snail species and persist across multiple generations. Our study also revealed a reduced gut microbial diversity in snail offspring, particularly in terms of unique gut bacteria. In successive generations of snail offspring, we observed a gradual increase in the abundance of the shared microbe *Aeromonas*, which differed from the pattern observed for the shared microbe *Cloacibacterium*. Additionally, we found that some bacteria shared among the offspring were also present in the cultured snails. Previous studies have shown that beneficial interactions and strain variability help maintain core gut microbial communities [42]. Therefore, the abundance of core microbes such as *Aeromonas* in snail offspring may benefit from the decrease in unique bacteria. However, the trends observed for other core microbes, such as *Cloacibacterium*, are different and show high relative abundance in different generations. Further studies are needed to understand the evolutionary aspects of gut microbiota, especially core microbes, across multiple generations in snails.

In freshwater gastropods, core gut microbes were found to be associated with various developmental stages. The gut microbiota in mammals is influenced by various factors [53]. For example, studies have revealed that the genera *Prevotella*, *Bacteroides*, and Ruminococcaceae are the three dominant gut taxa in humans [61–63], although their abundances differ in mice [64, 65]. Rats, on the other hand, harbored diverse gut bacteria including *Lactobacillus* and Enterobacteriaceae [66]. The gut microbiota of rhesus macaques contains *Prevotella*, *Campylobacter*, and Lachnospiraceae, and the relative abundance of these gut microbes is related to age [67]. These mammals shared one or more microbes as the dominant gut taxa. Similarly, African cichlid fishes and zebrafish [25, 68, 69] have *Cetobacterium* as the most prevalent genus in their gut microbiota, with diet variety playing a role in maintaining the core gut microbial communities [42], The microbes *Pseudomonas*, *Pseudoalteromonas*, and *Luteibacter* were dominant bacterial genera in the midgut of *Ae. aegypti* [56]. Previous studies have revealed that the phylum Bacteroidetes and genus *Fluviicola* were the dominant bacteria in *B*. *glabrata* [18], while others [70] found that *Aeromonas*, *Citrobacter*, and *Enterobacter* were the dominant genera in *B. glabrata*. Although *B. straminea* and *B. glabrata* belong to the same genus, their microbiota community composition is different. In gastropods, the strain, abundance, and diversity of core microbes may be influenced by host genetics, diet, habitat, and developmental stage. However, the distribution of “core” microbes in the gut of gastropods across developmental stages is unclear.

Previous studies have classified gut microbes appearing at certain stages during longitudinal development as “passengers,” while those persistently present from birth to adulthood are called “residents” [43]. Our study first revealed that the genera *Aeromonas* and *Cloacibacterium* are persistently present throughout the lifespan of snails and are detected in all developmental stages. However, other bacterial gut residents, such as the dominant genera unidentified *Rhizobiaceae*, only appeared at certain stages or developmental time points, indicating a mode of horizontal transmission [14]. Currently, there is limited research on longitudinal investigations of gastropod gut flora at different developmental stages. Previous studies focused on describing the gut microbiotas of adult snails, including the diversity, richness, and predicted metabolic functions [70–75]. Our study first examined the microbiota in *B. straminea* throughout its life cycle, including maternal, egg, youth, adult, and older stages. We observed an overall increasing trend in bacterial alpha diversity across different developmental stages in the gastropods, which aligned with previous findings in the gut microbiome of swine [43, 76]. In this study, the gastropod gut microbiota of older-stage gastropods showed the highest relative abundance among developmental stages, possibly due to environmental factors [53]. Our results indicated that core microbes were associated with specific gastropod developmental stages and may play critical roles in host physiology and development. We also found that the gut bacteria *Aeromonas* and *Cloacibacterium* in offspring during select stages of development could be traced to maternal snails, confirming the vertical transmission and longitudinal distribution of core microbes during the life cycle of gastropods. Our findings shed light on the persistent transmission of specific gut microbes throughout gastropod generations and life cycles. Overall, our findings highlight the persistent transmission of specific gut microbes throughout gastropod generations and life cycles, suggesting their potential importance in host physiological processes.

Our research provides evidence for the potential development of strategies aimed at controlling invasive gastropods through the manipulation of gut microbes. Pathogenic microorganisms that cause harm to the host by enhancing virulence are mostly transmitted horizontally [14, 77, 78]. Dysbacteriosis of the gut microbiota has previously been shown to lead to sickness or death in hosts [79, 80]. Invasive alien species (IAS) may pose a significant threat to both human health and national security [6, 8], with the absence of efficient natural predators being one of the key issues faced by control strategies for IAS [8, 81]. Biocontrol remains a hopeful strategy for controlling invasive species in ways that are friendly to the environment [82, 83]. Our findings suggest that disturbing the homeostasis of the gut microbiota to identify the potential pathogenic microbes that are correlated with host nonadaptation could serve as a potential strategy for controlling invasive snails. In addition, previous studies have revealed that certain gut bacteria found in mosquitoes, such as *Serratia* bacterium strain (AS1) [84] and *Serratia ureilytica* (Su_YN1) [85], could be potential weapons for blocking malaria transmission. These gut microbiota can disseminate through mosquito populations by vertical and horizontal transmission. Similarly, the common core gut microbes *Aeromonas* and *Cloacibacterium* found in freshwater gastropods, which serve as intermediate hosts of schistosomes, can also be vertically and horizontally transmitted. However, the relationship between these core microbes and the biological aspects of their hosts requires further study.

Whether the loss of diversity in gut microbiome in snail offspring is associated with environments or diets is unclear. Firstly, *Aeromonas* and *Cloacibacterium* are bacteria originally found in aquatic environments [86–89]. Environment plays an important role in the sources of gut microbiota in organisms [41, 90–92]. Mosquitoes acquire most gut bacteria during the larval stage, which is associated with the water-environmental microbes [41]. Secondly, previous studies have revealed that diet can significantly affect the composition and structures of gut microbiota in hosts [93–95]. Our previous study has shown that diet change can decrease the diversity of snail gut microbiota [96]. Last but not the least, the snails reared under laboratory conditions were maintained with sterile food. In total, although the relationship between gastropod gut microbiota and environmental bacteria is not well-studied, we thought that microorganisms in the environments may be important factors affecting the changes of gut microbiota in offspring snails. The sources of snail gut microbiota may provide a basis for the understanding of the biological characteristics of gastropods.

Finally, *P. canaliculata*, *B. straminea*, and *Achatina fulica* are significant invasive snails that play a crucial role in transmitting parasites such as *A. cantonensis* or *S. mansoni*, thereby contributing to the dissemination of infectious diseases [97]. As a result, it is crucial to continuously monitor the spread of these invasive snails and implement effective control strategies. However, due to environmental concerns, high toxicity to nontarget animals, and exorbitant costs, chemical molluscicides are not widely used [5, 98]. Consequently, there is an urgent need for low-toxicity and environmentally friendly tools to control parasite-transmitting snails. Biological control, which involves the use of specific gut microbiota or microorganisms, has emerged as a promising approach for the management of IAS and pathogens [5, 84, 99–101]. This study revealed the modes of microbial transmission in the guts of gastropod mollusks and showed that core gut microbes *Cloacibacterium* and *Aeromonas* associated with different gastropod species could be persistently transmitted across multiple generations. Similarly, the gut microbiota of mosquitoes, including the AS1 [84] and Su_YN1 [85], can be horizontally transmitted through mating and vertically transmitted by adhering to eggshells, offering a promising approach to combat diseases like malaria. Therefore, investigating the transmission mode of gut microbiota in vector snails may provide valuable insights into the distribution and transmission patterns of important gut microbes. This knowledge has the potential to enhance our understanding and control of snail-borne diseases by targeting intermediate hosts, presenting a novel and environmentally friendly approach for prevention and control. Nevertheless, further research is required to fully explore these possibilities.

## Conclusions

We conducted a long-term and multigenerational experiment to reveal the transmission routes of the gut microbiota in gastropods from wild populations and cultured generations. Our results revealed several important findings:

- The gut microbiota of wild and cultured gastropods differ significantly, with the core gut microbes *Aeromonas* and *Cloacibacterium* associated with different gastropod species.
- Horizontal transmission accounted for the majority of gut microbiota transmission in gastropods, but less than 50% of the gut microbiota could be vertically transmitted in a nonsterile environment, on average.
- Under nonsterile conditions, the wild gastropod gut microbiota could be maintained for up to 2 generations, after which the gut microbiomes in the F3 and F4 generations became similar to those of cultured snails.
- The convergent evolution of the gut microbiota may contribute to the adaptive evolution of gastropods from wild to cultured environments, and this was associated with metabolic functions.
- Vertical transmission, rather than horizontal transmission, drove the convergent evolution of the gut microbiota, promoting host health. This suggests that shifts in the gut microbiota community structure can serve as potential indicators of the level or current status of host health.
- The core microbes *Aeromonas* and *Cloacibacterium* were found to be vertically transmitted across multiple generations and longitudinal transmitted across developmental stages.

This study provides novel insights into the dynamic process of gut microbiota development during adaptive evolution and across host developmental stages. Furthermore, it experimentally reveals the transmission modes of gut microbiota in gastropods.

## Materials and methods

### Sample collection and study design

To investigate the differences in the gut microbiota between wild and cultured freshwater gastropods, field studies were conducted. Adult snail species including *B. straminea*, *B. aeruginosa*, *P. acuta*, *P. corneus*, and *P. canaliculata* were collected from various sites in Hong Kong, Guangdong, and Guangxi in southern China between 2017 and 2018. A total of 44 wild snail samples were collected from Guangxi, Guangdong, and Hong Kong (Fig. S11), and the number of collected snails was counted. The geographic coordinates of all surveyed sites were also documented using a handheld Global Positioning System (GPS) device. Upon collection, the wild snails were transferred to the laboratory and immediately dissected.

Cultured snails including *B. straminea*, *B. glabrata*, *B. aeruginosa*, *B. pfeifferi*,* P. acuta*, and *P. corneus* were also collected for comparison. Cultured *B. straminea* and *P. acuta* were obtained from Shenzhen, China, and had been maintained in laboratory conditions for over 10 years. Cultured *B. glabrata* was introduced from the University of Bristol School of Veterinary Sciences in the UK and had also been reared in laboratory conditions for over 10 years. Cultured *B. aeruginosa* snails were collected from Guangzhou, China, more than 8 years ago and subsequently cultured in the laboratory.

To investigate the long-term shift processes and transmission modes of gut microbiota in gastropods, we transferred wild gastropod snails into the laboratory environment and reared them under controlled conditions. Gut samples were immediately collected from these wild snails, as well as from their offspring over multiple generations. We detected shifts in the gut microbiota in wild gastropods after transfer and analyzed the evolution of the gut microbiomes in subsequent generations of wild snail offspring. Finally, we compared the differences in gut microbiota among wild snails, their offspring, and cultured populations.

### Snail husbandry and identification

Wild-caught snails collected from southern China were transferred to the Department of Parasitology at Sun Yat-sen University. Benjamin Sanogo, a researcher from Mali, provided us with DNA samples of *B. pfeifferi*. The snails were fed sterile food that was obtained through autoclave sterilization. They were housed in an aquatic tank filled with dechlorinated tap water, which was replaced every 2 days. The temperature was maintained at 25 to 27 °C with 80% relative humidity (RH) and a 12:12 h (L:D) light cycle. We monitored the health of the snails and their offspring by assessing the survival rate and mortality. Morphological observation of the wild snails was performed.

The identification of snail species relies primarily on morphological characteristics, and molecular biology techniques are used as necessary. The molecular identification is based on the *CO1* gene to identify snail species. To extract total DNA, we used the HiPure DNA Mini Kit (Magen, China) on the head-foot tissue of the snail. The *CO1* gene primer set (forward: 5′-GGTCAACAAATCATAAAGATATTGG-3′, reverse: 5′-TAAACTTCAGGGTGACCAAAAAATCA-3′) was employed for PCR amplification. The PCR products were evaluated using agarose gel electrophoresis (1.5% gel) and purified according to the manufacturer’s instructions with the agarose gel DNA purification kit (TaKaRa, China). The PCR conditions were as follows: initial denaturation at 94 °C for 5 min, followed by 30 cycles of 94 °C for 50 s, annealing at 55 °C for 50 s, extension at 72 °C for 50 s, and a final extension at 72 °C for 10 min. The obtained *CO1* sequences were aligned and concatenated with sequences from the National Center for Biotechnology Information (NCBI) databases (https://www.ncbi.nlm.nih.gov/). The alignment was performed using the neighbor-joining method with 1500 nonparametric bootstrap replicates in MEGA7 (version 7.0.18) [102].

### Intestine isolation

To isolate the intestine, the whole snail was surface washed in sterile water for 30 s and washed three times in separate sterile dishes. Subsequently, the snails were rinsed in sterile phosphate-buffered saline (PBS) for 15 s. The shell was removed to access the body tissue of the snail (Fig. S12), which was then rinsed twice with sterile PBS for 15 s. After this procedure, the body was placed in a sterile dish and dissected to isolate the intestine. All dissections and sample collections were conducted under consistent sterile conditions. The collected samples were stored at − 80 °C for further study.

### DNA extraction, amplification, library preparation, and sequencing of 16S rRNA genes

Each intestinal sample from the snails comprised either one gut or two guts. Each egg sample included 3 egg masses. The gut sample was homogenized into suspension using a tissue homogenizer. Total DNA was then extracted from both egg and gut samples using the HiPure Bacterial DNA Kit protocol (Magen, China). The quality and quantity of the extracted DNA were examined using a NanoDrop (Thermo Scientific, USA). The total genomic DNA was then suspended in 30-μl nuclease-free buffer and stored at − 80 °C until further studies. The concentration and purity of the total DNA were monitored on 1% agarose gel electrophoresis.

Next, the hypervariable region (V3–V4) of 16S rRNA genes was amplified for each sample using universal-specific primers. The bacterial 341F/806R primer sets were used: forward 5′-CCTAYGGGRBGCASCAG-3′ and reverse 5′-GGACTACNNGGGTATCTAAT-3′ [103]. The PCR cycling conditions included an initial denaturation step at 98 °C for 1 min, followed by 30 cycles of 98 °C for 10 s, 50 °C for 30 s, and 72 °C for 30 s, with a final extension step at 72 °C for 5 min. A single PCR was made for each sample. The PCR products were visualized on 2% agarose gel electrophoresis and purified based on the manufacturer’s instructions for the Qiagen Gel Extraction Kit (Qiagen, Germany).

Sequencing libraries for 16S rRNA gene sequencing were generated according to the TruSeq DNA PCR-Free Sample Preparation Kit protocol (Illumina, USA). The library quality was assessed on the Qubit 2.0 Fluorometer (Thermo Scientific) and Agilent Bioanalyzer 2100 system. The V3–V4 regions of the 16S rRNA gene library were then sequenced on an Illumina HiSep PE250 platform constructed by Novogene (Beijing, China), generating paired-end reads.

### Bacterial data analysis

To analyze the bacterial data, sequence assembly [104], data filtration [105, 106], and chimera removal steps [107, 108] were performed to obtain effective sequences. The raw 16S rRNA sequences were processed using QIIME (version 1.9.1). Sequences with 97% or greater similarity were assigned to the same OTUs using UPARSE software (v. 7.0.1001) [109]. The SILVA database (v. 138.1) was used to identify each representative sequence [110]. Rare reads were removed. MUSCLE software (v. 3.8.31) was used to analyze the phylogenetic relationship construction among different OTUs. Alpha and beta diversities were determined based on the abundance of OTUs. Alpha-diversity indices, such as Shannon index and observed species index, were calculated to assess community richness using QIIME (v. 1.9.1) and displayed with R software (v. 2.15.3). Rarefaction curve analysis, Venn diagram analysis, and ridgeline analysis were performed using R software (v. 2.15.3). Beta diversity was calculated to evaluate differences among samples using QIIME (v. 1.9.1) and displayed with R software (v. 2.15.3). Principal coordinate analysis (PCoA) and principal component analysis (PCA) based on binary Jaccard distance were performed to obtain principal coordinates and visualize complex, multidimensional data in R (v. 2.15.3). Microbiome similarities were identified using the analysis of similarity (ANOSIM) method. Linear discriminant analysis effect size (LEfSe) was used to generate an LDA effect size in LEfSe (v. 1.0). Network analysis was performed in GraphViz software (v. 2.38.0), while random forest models were developed using the R (v. 2.15.3) to identify bacterial features. Correlation network analysis was conducted using Cytoscape (v. 3.9.0) [111]. Additionally, the relative abundance of metabolic functions in the gut microbiota of gastropods was analyzed using the FAPROTAX database (v. 1.14) at the OTU level. The Wilcoxon test was used for pairwise comparisons, with statistical significance defined at *P* < 0.05.

### Statistical analysis

The data were expressed as the mean ± standard error of the mean (SEM) and analyzed to calculate the relationship between groups using GraphPad Prism (v. 5.0; GraphPad Software, USA). Differences between groups were assessed using *t*-tests performed using SPSS 19.0 software (SPSS Inc., USA). *P* < 0.05 was considered statistically significant.

### Supplementary Information

**Additional file 1:** **Table S1.** Information of sequenced samples from wild and cultured gastropods. **Table S2.** Details of samples collected from wild gastropods and and the translocated snails in this study. **Table S3.** Gut microbial community dissimilarities of Wild and WildT *B. straminea *cultured by analysis of similarity (ANOSIM). *R*-value is greater than 0, showing significant differences between groups. The credibility of statistical analysis is represented by the *P*-value. **Table S4.** Details of samples collected from wild gastropods, their offspring and cultured gastropods in this study. **Table S5.** Gut microbial community dissimilarities of wild *B. straminea* and their offspring over generations demonstrated by analysis of similarity (ANOSIM). *R*-value is greater than 0, showing differences between groups. The credibility of statistical analysis is represented by the *P*-value. **Table S6.** Details of samples collected from offspring of wild gastropods and cultured gastropods in this study. **Table S7.** Summary of nodes and edges (positive and negative) for each group or taxon. **Table S8.** Details of gastropod samples collected through important life stages in this study. **Table S9.** Gut microbial community dissimilarities of gastropod B. straminea through important life stages demonstrated by ANOSIM. *R*-value is greater than 0, showing significant differences between groups. The credibility of statistical analysis is represented by the *P*-value.

**Additional file 2: Fig. S1.** Overview of the gut microbiome of freshwater gastropods at the genus (Top 50) and phylum level. The yellow and red columns indicated the relative abundance of gut microbes of wild and cultured populations, respectively. Cultured snails refer to the laboratory snails cultured under laboratory conditions. **Fig. S2.** Overview of the gut microbiome of freshwater gastropods at the genus and phylum level. a-f Gut bacterial genera of cultured freshwater gastropods, including B. straminea (*n* =6), B. aeruginosa (*n* =8), P. acuta (*n* =4), P. corneus (*n* =6), B. glabrata (*n* =7) and B. pfeif eri (*n* =8). g-k Gut bacterial genera of wild-caught freshwater snails, including P. canaliculata (*n* =3), B. straminea (*n* =14), B. aeruginosa (*n* =6), P. acuta (*n* =6) and P. corneus (*n* =6). l The relative abundance of bacterial phyla. Cultured snails refer to the laboratory snails cultured under laboratory conditions. **Fig. 3.** Difference in gut microbiota between wild and cultured freshwater gastropods. a At the genus level. b A linear discriminant analysis (LDA) effect size (LEfSe) analysis. Cultured snails refer to the laboratory snails cultured under laboratory conditions. **Fig. S4.** The relative abundance of gut microbiota of Wild and Wildt gastropods. Wild: wild-caught snails. WildT: wild-caught snails being transferred to the laboratory. **Fig. S5.** Overview of vertical shifts of gut microbes in Planorbarius corneus gastropods over generations. a PCoA of gut microbiota communities of wild and the F1 generation of Planorbarius corneus snails. b PCoA. c The number of OTUs are shared or unique to sample types. PlCmat: Maternal P. corneus snails. PlCoffsp: the offspring (F1 genernation) of maternal P. corneus snails reared under laboratory conditions. **Fig. S6.** Longitudinal shifts (from 0 days to 450 days) in the gut microbiome community diversity of freshwater snails. a Shannon index. b Observed species in the gut microbiome community diversity of freshwater snails. a Shannon index. b Observed species index. **Fig. S7.** The relative abundance of gut microbiota of freshwater gastropods across developmental stages. a At the phylum level. b At the family level. c At the genus level. **Fig. S8.** Differential gut bacterial taxa analyzed by LEfSe analysis with LDA score >4 across developmental stages. **Fig. S9.** a Percentage of OTUs from maternal snails transmitted to eggs. b Percentage of OTUs from maternal snails transmitted to snails at different developmental stages. c Venn diagram of the “core” microbes shared among different developmental stages. **Fig. S10.** Adaptation of gastropods is associated with gut microbiota. a Shannon index. b UPGMA analysis conducted based on binary Jaccard distance. c Phylogenetic evolutionary constructed based on the maximum-likelihood algorithm for CO1 gastropod sequences. d-g Top 30 fitness-related OTUs compared with different local populations are shown. **Fig. S11.** Overview of sampling study of wild snails. a Wild snails were collected from Guangdong (b), Guangxi (c), and Hong Kong (d) in China. The major sites were marked on the maps. The red triangles indicate the sampling sites. **Fig. S12.** Picture of body tissue of Biomphalaria straminea.

## Data Availability

All data generated or analyzed during this study are included in this published article and its supplementary information files.

## Abbreviations

OTU: Operational taxonomic unit
PCoA: Principal coordinate analysis
PCA: Principal component analysis
ANOSIM: Analysis of similarities
UPGMA: Unweighted pair group produced with arithmetic mean
LDA: Linear discriminant analysis
LEfSe: Linear discriminant analysis effect size
PCR: Polymerase chain reaction
AUC: Area under the curve
ROC: Receiver operating characteristic curve
CO1: Cytochrome c oxidase subunit I
F: Filial generation
IAS: Invasive alien species
RH: Relative humidity
PBS: Phosphate-buffered saline
GPS: Global Positioning System

## References

[1] Fan Y, Pedersen O (2021). Gut microbiota in human metabolic health and disease. Nat Rev Microbiol.

[2] Rosshart SP, Vassallo BG, Angeletti D, Hutchinson DS, Morgan AP, Takeda K (2017). Wild mouse gut microbiota promotes host fitness and improves disease resistance. Cell.

[3] Lin D, Zeng X, Sanogo B, He P, Xiang S, Du S (2020). The potential risk of *Schistosoma mansoni* transmission by the invasive freshwater snail *Biomphalaria straminea* in South China. PLoS Negl Trop Dis.

[4] Meier-Brook C (1974). A snail intermediate host of *Schistosoma mansoni* introduced into Hong Kong. Bull World Health Organ.

[5] Lin D, Xiang S, Sanogo B, Liang Y, Sun X, Wu Z (2021). Molecular characterization of rotifers and their potential use in the biological control of *Biomphalaria*. Front Cell Infect Microbiol.

[6] Tay WT, Gordon K (2019). Going global - genomic insights into insect invasions. Curr Opin Insect Sci.

[7] French NP, Gemmell NJ, Buddle BM (2007). Advances in biosecurity to 2010 and beyond: towards integrated detection, analysis and response to exotic pest invasions. N Z Vet J.

[8] Lester PJ, Beggs JR (2019). Invasion success and management strategies for social Vespula wasps. Annu Rev Entomol.

[9] Guo YH, Lv S, Gu WB, Liu HX, Wu Y, Zhang Y (2014). Investigation on the species distribution and infection status of host snails of *Angiostrongylus cantonensis* in Shanghai (In Chinese). Zhongguo Ji Sheng Chong Xue Yu Ji Sheng Chong Bing Za Zhi.

[10] Tomaz TP, Gentile R, Garcia JS, Teixeira BR, Faro MJ (2018). A survey of freshwater and terrestrial snails in a predominantly urban municipality of Rio de Janeiro State, Brazil, with emphasis on human parasites vectors. Rev Inst Med Trop Sao Paulo.

[11] Ji L, Yiyue X, Xujin H, Minghui Z, Mengying Z, Yue H (2017). Study on the tolerance and adaptation of rats to *Angiostrongylus cantonensis* infection. Parasitol Res.

[12] Lozupone CA, Stombaugh JI, Gordon JI, Jansson JK, Knight R (2012). Diversity, stability and resilience of the human gut microbiota. Nature.

[13] Diaz HR, Wang S, Anuar F, Qian Y, Bjorkholm B, Samuelsson A (2011). Normal gut microbiota modulates brain development and behavior. Proc Natl Acad Sci U S A.

[14] Moeller AH, Suzuki TA, Phifer-Rixey M, Nachman MW (2018). Transmission modes of the mammalian gut microbiota. Science.

[15] Vannier N, Mony C, Bittebiere AK, Michon-Coudouel S, Biget M, Vandenkoornhuyse P (2018). A microorganisms’ journey between plant generations. Microbiome.

[16] Chalifour B, Li J (2021). Characterization of the gut microbiome in wild rocky mountainsnails (*Oreohelix strigosa*). Anim Microbiome.

[17] Kivistik C, Knobloch J, Kairo K, Tammert H, Kisand V, Hildebrandt JP (2020). Impact of salinity on the gastrointestinal bacterial community of Theodoxus fluviatilis. Front Microbiol.

[18] Osorio JB, de Mattos PL, Giongo A, Marconatto L, Potriquet J, Candido R (2020). Mollusk microbiota shift during *Angiostrongylus cantonensis* infection in the freshwater snail *Biomphalaria glabrata* and the terrestrial slug *Phillocaulis soleiformis*. Parasitol Res.

[19] Van Horn DJ, Garcia JR, Loker ES, Mitchell KR, Mkoji GM, Adema CM (2012). Complex intestinal bacterial communities in three species of planorbid snails. J Molluscan Stud.

[20] Liu C, Zhang Y, Ren Y, Wang H, Li S, Jiang F, et al. The genome of the golden apple snail *Pomacea canaliculata* provides insight into stress tolerance and invasive adaptation. Gigascience. 2018;7(9):1–13. 10.1093/gigascience/giy101 PMC6129957 30107526

[21] Guo Y, Zhang Y, Liu Q, Huang Y, Mao G, Yue Z, et al. A chromosomal-level genome assembly for the giant African snail *Achatina fulica*. Gigascience. 2019;8(10):1–8. 10.1093/gigascience/giz124 PMC6802634 31634388

[22] Henning F, Meyer A (2014). The evolutionary genomics of cichlid fishes: explosive speciation and adaptation in the postgenomic era. Annu Rev Genomics Hum Genet.

[23] Muschick M, Indermaur A, Salzburger W (2012). Convergent evolution within an adaptive radiation of cichlid fishes. Curr Biol.

[24] Alejandrino A, Puslednik L, Serb JM (2011). Convergent and parallel evolution in life habit of the scallops (Bivalvia: Pectinidae). BMC Evol Biol.

[25] Baldo L, Pretus JL, Riera JL, Musilova Z, Bitja NA, Salzburger W (2017). Convergence of gut microbiotas in the adaptive radiations of African cichlid fishes. ISME J.

[26] Zhang Z, Xu D, Wang L, Hao J, Wang J, Zhou X (2016). Convergent evolution of rumen microbiomes in high-altitude mammals. Curr Biol.

[27] Moeller AH, Sanders JG (1808). Roles of the gut microbiota in the adaptive evolution of mammalian species. Philos Trans R Soc Lond B Biol Sci.

[28] Dvergedal H, Sandve SR, Angell IL, Klemetsdal G, Rudi K (2020). Association of gut microbiota with metabolism in juvenile Atlantic salmon. Microbiome.

[29] Zhang J, Liu YX, Zhang N, Hu B, Jin T, Xu H (2019). NRT1.1B is associated with root microbiota composition and nitrogen use in field-grown rice. Nat Biotechnol..

[30] Browne HP, Neville BA, Forster SC, Lawley TD (2017). Transmission of the gut microbiota: spreading of health. Nat Rev Microbiol.

[31] Li F, Li C, Chen Y, Liu J, Zhang C, Irving B (2019). Host genetics influence the rumen microbiota and heritable rumen microbial features associate with feed efficiency in cattle. Microbiome.

[32] Portet A, Toulza E, Lokmer A, Huot C, Duval D, Galinier R, et al. Experimental infection of the *Biomphalaria glabrata* vector snail by *Schistosoma mansoni* parasites drives snail microbiota dysbiosis. Microorganisms. 2021;9(5):1084. 10.3390/microorganisms9051084 PMC8158356 34070104

[33] Li H, Hambrook JR, Pila EA, Gharamah AA, Fang J, Wu X, et al. Coordination of humoral immune factors dictates compatibility between *Schistosoma mansoni* and *Biomphalaria glabrata*. Elife. 2020;9:e51708. 10.7554/eLife.51708 PMC6970513 31916937

[34] Adema CM, Hillier LW, Jones CS, Loker ES, Knight M, Minx P (2017). Whole genome analysis of a schistosomiasis-transmitting freshwater snail. Nat Commun.

[35] Dejong RJ, Morgan JA, Paraense WL, Pointier JP, Amarista M, Ayeh-Kumi PF (2001). Evolutionary relationships and biogeography of *Biomphalaria* (Gastropoda: Planorbidae) with implications regarding its role as host of the human bloodfluke. Schistosoma mansoni Mol Biol Evol.

[36] Fernandez MA, Thiengo SC (2010). Susceptibility of *Biomphalaria straminea* from Peixe Angical dam, Tocantins, Brazil to infection with three strains of *Schistosoma mansoni*. Mem Inst Oswaldo Cruz.

[37] Fernandez MA, Pieri OS (2001). Infection by *Schistosoma mansoni* Sambon 1907 in the first four months of life of *Biomphalaria straminea* (Dunker, 1848) in Brazil. Mem Inst Oswaldo Cruz.

[38] Gandasegui J, Fernandez-Soto P, Muro A, Simoes BC, Lopes DMF, Loyo R (2018). A field survey using LAMP assay for detection of *Schistosoma mansoni* in a low-transmission area of schistosomiasis in Umbuzeiro, Brazil: assessment in human and snail samples. PLoS Negl Trop Dis.

[39] Rani A, Sharma A, Rajagopal R, Adak T, Bhatnagar RK (2009). Bacterial diversity analysis of larvae and adult midgut microflora using culture-dependent and culture-independent methods in lab-reared and field-collected *Anopheles stephensi*-an Asian malarial vector. BMC Microbiol.

[40] Lin D, Zheng X, Sanogo B, Ding T, Sun X, Wu Z (2021). Bacterial composition of midgut and entire body of laboratory colonies of *Aedes aegypti* and *Aedes albopictus* from southern China. Parasit Vectors.

[41] Bascunan P, Nino-Garcia JP, Galeano-Castaneda Y, Serre D, Correa MM (2018). Factors shaping the gut bacterial community assembly in two main Colombian malaria vectors. Microbiome.

[42] Kokou F, Sasson G, Friedman J, Eyal S, Ovadia O, Harpaz S (2019). Core gut microbial communities are maintained by beneficial interactions and strain variability in fish. Nat Microbiol.

[43] Wang X, Tsai T, Deng F, Wei X, Chai J, Knapp J (2019). Longitudinal investigation of the swine gut microbiome from birth to market reveals stage and growth performance associated bacteria. Microbiome.

[44] Lin D, Song Q, Zhang Y, Liu J, Chen F, Du S, et al. *Bacillus subtilis* attenuates hepatic and intestinal injuries and modulates gut microbiota and gene expression profiles in mice infected with *Schistosoma japonicum*. Front Cell Dev Biol. 2021;9:766205. 10.3389/fcell.2021.766205 PMC8635066 34869360

[45] Li LH, Lv S, Lu Y, Bi DQ, Guo YH, Wu JT (2019). Spatial structure of the microbiome in the gut of *Pomacea canaliculata*. BMC Microbiol.

[46] Hu Z, Tong Q, Chang J, Yu J, Li S, Niu H (2021). Gut bacterial communities in the freshwater snail *Planorbella trivolvis* and their modification by a non-herbivorous diet. PeerJ.

[47] Kort R, Caspers M, van de Graaf A, van Egmond W, Keijser B, Roeselers G (2014). Shaping the oral microbiota through intimate kissing. Microbiome.

[48] Maqsood R, Rodgers R, Rodriguez C, Handley SA, Ndao IM, Tarr PI (2019). Discordant transmission of bacteria and viruses from mothers to babies at birth. Microbiome.

[49] Asnicar F, Manara S, Zolfo M, Truong DT, Scholz M, Armanini F, et al. Studying vertical microbiome transmission from mothers to infants by strain-level metagenomic profiling. mSystems. 2017;2(1):e00164–16. 10.1128/mSystems.00164-16 PMC5264247 28144631

[50] Ferretti P, Pasolli E, Tett A, Asnicar F, Gorfer V, Fedi S (2018). Mother-to-infant microbial transmission from different body sites shapes the developing infant gut microbiome. Cell Host Microbe.

[51] Backhed F, Roswall J, Peng Y, Feng Q, Jia H, Kovatcheva-Datchary P (2015). Dynamics and stabilization of the human gut microbiome during the first year of life. Cell Host Microbe.

[52] Avershina E, Lundgard K, Sekelja M, Dotterud C, Storro O, Oien T (2016). Transition from infant- to adult-like gut microbiota. Environ Microbiol.

[53] Rothschild D, Weissbrod O, Barkan E, Kurilshikov A, Korem T, Zeevi D (2018). Environment dominates over host genetics in shaping human gut microbiota. Nature.

[54] Delsuc F, Metcalf JL, Wegener PL, Song SJ, Gonzalez A, Knight R (2014). Convergence of gut microbiomes in myrmecophagous mammals. Mol Ecol.

[55] Song SJ, Sanders JG, Delsuc F, Metcalf J, Amato K, Taylor MW, et al. Comparative analyses of vertebrate gut microbiomes reveal convergence between birds and bats. mBio. 2020;11(1):e02901–19. 10.1128/mBio.02901-19 PMC6946802 31911491

[56] Dickson LB, Ghozlane A, Volant S, Bouchier C, Ma L, Vega-Rua A (2018). Diverse laboratory colonies of *Aedes aegypti* harbor the same adult midgut bacterial microbiome. Parasit Vectors.

[57] Aylward FO, Suen G, Biedermann PH, Adams AS, Scott JJ, Malfatti SA, et al. Convergent bacterial microbiotas in the fungal agricultural systems of insects. mBio. 2014;5(6):e2077. 10.1128/mBio.02077-14 PMC4251994 25406380

[58] Davenport ER, Sanders JG, Song SJ, Amato KR, Clark AG, Knight R (2017). The human microbiome in evolution. BMC Biol.

[59] Rosenberg E, Zilber-Rosenberg I (2018). The hologenome concept of evolution after 10 years. Microbiome.

[60] Moeller AH, Li Y, Mpoudi NE, Ahuka-Mundeke S, Lonsdorf EV, Pusey AE (2014). Rapid changes in the gut microbiome during human evolution. Proc Natl Acad Sci U S A.

[61] Ding T, Schloss PD (2014). Dynamics and associations of microbial community types across the human body. Nature.

[62] Falony G, Joossens M, Vieira-Silva S, Wang J, Darzi Y, Faust K (2016). Population-level analysis of gut microbiome variation. Science.

[63] Costea PI, Hildebrand F, Arumugam M, Backhed F, Blaser MJ, Bushman FD (2018). Enterotypes in the landscape of gut microbial community composition. Nat Microbiol.

[64] Cho I, Yamanishi S, Cox L, Methe BA, Zavadil J, Li K (2012). Antibiotics in early life alter the murine colonic microbiome and adiposity. Nature.

[65] Tran T, Cousin FJ, Lynch DB, Menon R, Brulc J, Brown JR (2019). Prebiotic supplementation in frail older people affects specific gut microbiota taxa but not global diversity. Microbiome.

[66] Yip LY, Aw CC, Lee SH, Hong YS, Ku HC, Xu WH (2018). The liver-gut microbiota axis modulates hepatotoxicity of tacrine in the rat. Hepatology.

[67] Rhoades N, Barr T, Hendrickson S, Prongay K, Haertel A, Gill L (2019). Maturation of the infant rhesus macaque gut microbiome and its role in the development of diarrheal disease. Genome Biol.

[68] Stephens WZ, Burns AR, Stagaman K, Wong S, Rawls JF, Guillemin K (2016). The composition of the zebrafish intestinal microbial community varies across development. ISME J.

[69] Kim PS, Shin NR, Lee JB, Kim MS, Whon TW, Hyun DW (2021). Host habitat is the major determinant of the gut microbiome of fish. Microbiome.

[70] Silva TM, Melo ES, Lopes AC, Veras DL, Duarte CR, Alves LC (2013). Characterization of the bacterial microbiota of *Biomphalaria glabrata* (Say, 1818) (Mollusca: Gastropoda) from Brazil. Lett Appl Microbiol.

[71] Cardoso AM, Cavalcante JJ, Cantao ME, Thompson CE, Flatschart RB, Glogauer A (2012). Metagenomic analysis of the microbiota from the crop of an invasive snail reveals a rich reservoir of novel genes. PLoS ONE.

[72] Cardoso AM, Cavalcante JJ, Vieira RP, Lima JL, Grieco MA, Clementino MM (2012). Gut bacterial communities in the giant land snail *Achatina fulica* and their modification by sugarcane-based diet. PLoS ONE.

[73] Charrier M, Fonty G, Gaillard-Martinie B, Ainouche K, Andant G (2006). Isolation and characterization of cultivable fermentative bacteria from the intestine of two edible snails, *Helixpomatia* and *Cornu aspersum* (Gastropoda: Pulmonata). Biol Res.

[74] Takacs-Vesbach C, King K, Van Horn D, Larkin K, Neiman M (2016). Distinct bacterial microbiomes in sexual and asexual *Potamopyrgus antipodarum*, a New Zealand freshwater snail. PLoS ONE.

[75] Huot C, Clerissi C, Gourbal B, Galinier R, Duval D, Toulza E (2019). Schistosomiasis vector snails and their microbiota display a phylosymbiosis pattern. Front Microbiol.

[76] Lu D, Tiezzi F, Schillebeeckx C, Mcnulty NP, Schwab C, Shull C (2018). Host contributes to longitudinal diversity of fecal microbiota in swine selected for lean growth. Microbiome.

[77] Lipsitch M, Siller S, Nowak MA (1996). The evolution of virulence in pathogens with vertical and horizontal transmission. Evolution.

[78] Brown SP, Cornforth DM, Mideo N (2012). Evolution of virulence in opportunistic pathogens: generalism, plasticity, and control. Trends Microbiol.

[79] Atarashi K, Suda W, Luo C, Kawaguchi T, Motoo I, Narushima S (2017). Ectopic colonization of oral bacteria in the intestine drives TH1 cell induction and inflammation. Science.

[80] Videvall E, Song SJ, Bensch HM, Strandh M, Engelbrecht A, Serfontein N (2020). Early-life gut dysbiosis linked to juvenile mortality in ostriches. Microbiome.

[81] Graham S, Metcalf AL, Gill N, Niemiec R, Moreno C, Bach T (2019). Opportunities for better use of collective action theory in research and governance for invasive species management. Conserv Biol.

[82] Duval D, Galinier R, Mouahid G, Toulza E, Allienne JF, Portela J (2015). A novel bacterial pathogen of *Biomphalaria glabrata*: a potential weapon for schistosomiasis control?. PLoS Negl Trop Dis.

[83] Teem JL, Alphey L, Descamps S, Edgington MP, Edwards O, Gemmell N (2020). Genetic biocontrol for invasive species. Front Bioeng Biotechnol.

[84] Wang S, Dos-Santos A, Huang W, Liu KC, Oshaghi MA, Wei G (2017). Driving mosquito refractoriness to *Plasmodium falciparum* with engineered symbiotic bacteria. Science.

[85] Gao H, Bai L, Jiang Y, Huang W, Wang L, Li S (2021). A natural symbiotic bacterium drives mosquito refractoriness to *Plasmodium* infection via secretion of an antimalarial lipase. Nat Microbiol.

[86] Hua ZL, Wang YF, Zhang JY, Li XQ, Yu L. Removal of perfluoroalkyl acids and dynamic succession of biofilm microbial communities in the decomposition process of emergent macrophytes in wetlands. Sci Total Environ. 2022;834:155295. 10.1016/j.scitotenv.2022.155295 35439517

[87] Mendoza-Barbera E, Merino S, Tomas J. Surface glucan structures in *Aeromonas* spp. Mar Drugs. 2021;19(11):649. 10.3390/md19110649 PMC8625153 34822520

[88] Yu K, Li P, Chen Y, Zhang B, Huang Y, Huang FY (2020). Antibiotic resistome associated with microbial communities in an integrated wastewater reclamation system. Water Res.

[89] Carney RL, Labbate M, Siboni N, Tagg KA, Mitrovic SM, Seymour JR (2019). Urban beaches are environmental hotspots for antibiotic resistance following rainfall. Water Res.

[90] Chen CY, Chen CK, Chen YY, Fang A, Shaw GT, Hung CM (2020). Maternal gut microbes shape the early-life assembly of gut microbiota in passerine chicks via nests. Microbiome.

[91] Dominguez-Bello MG, De Jesus-Laboy KM, Shen N, Cox LM, Amir A, Gonzalez A (2016). Partial restoration of the microbiota of cesarean-born infants via vaginal microbial transfer. Nat Med.

[92] Moor J, Wuthrich T, Aebi S, Mostacci N, Overesch G, Oppliger A (2021). Influence of pig farming on human gut microbiota: role of airborne microbial communities. Gut Microbes.

[93] Muegge BD, Kuczynski J, Knights D, Clemente JC, Gonzalez A, Fontana L (2011). Diet drives convergence in gut microbiome functions across mammalian phylogeny and within humans. Science.

[94] Baniel A, Amato KR, Beehner JC, Bergman TJ, Mercer A, Perlman RF (2021). Seasonal shifts in the gut microbiome indicate plastic responses to diet in wild geladas. Microbiome.

[95] Kang W, Kim PS, Tak EJ, Sung H, Shin NR, Hyun DW (2022). Host phylogeny, habitat, and diet are main drivers of the cephalopod and mollusk gut microbiome. Anim Microbiome.

[96] Du S, Sun X, Zhang J, Lin D, Chen R, Cui Y (2022). Metagenome-assembled genomes reveal mechanisms of carbohydrate and nitrogen metabolism of schistosomiasis-transmitting vector *Biomphalaria glabrata*. Microbiol Spectr.

[97] Zhu GL, Tang YY, Limpanont Y, Wu ZD, Li J, Lv ZY (2019). Zoonotic parasites carried by invasive alien species in China. Infect Dis Poverty.

[98] Twining JP, Lawton C, White A, Sheehy E, Hobson K, Montgomery WI (2022). Restoring vertebrate predator populations can provide landscape-scale biological control of established invasive vertebrates: Insights from pine marten recovery in Europe. Glob Chang Biol.

[99] Zheng X, Zhang D, Li Y, Yang C, Wu Y, Liang X (2019). Incompatible and sterile insect techniques combined eliminate mosquitoes. Nature.

[100] Baldacchino F, Caputo B, Chandre F, Drago A, Della TA, Montarsi F (2015). Control methods against invasive *Aedes* mosquitoes in Europe: a review. Pest Manag Sci.

[101] Gao H, Cui C, Wang L, Jacobs-Lorena M, Wang S (2020). Mosquito microbiota and implications for disease control. Trends Parasitol.

[102] Kumar S, Stecher G, Tamura K (2016). MEGA7: Molecular Evolutionary Genetics Analysis version 7.0 for bigger datasets. Mol Biol Evol..

[103] Lin D, Song Q, Liu J, Chen F, Zhang Y, Wu Z (2022). Potential gut microbiota features for non-invasive detection of schistosomiasis. Front Immunol.

[104] Magoc T, Salzberg SL (2011). FLASH: fast length adjustment of short reads to improve genome assemblies. Bioinformatics.

[105] Bokulich NA, Subramanian S, Faith JJ, Gevers D, Gordon JI, Knight R (2013). Quality-filtering vastly improves diversity estimates from Illumina amplicon sequencing. Nat Methods.

[106] Caporaso JG, Kuczynski J, Stombaugh J, Bittinger K, Bushman FD, Costello EK (2010). QIIME allows analysis of high-throughput community sequencing data. Nat Methods.

[107] Edgar RC, Haas BJ, Clemente JC, Quince C, Knight R (2011). UCHIME improves sensitivity and speed of chimera detection. Bioinformatics.

[108] Haas BJ, Gevers D, Earl AM, Feldgarden M, Ward DV, Giannoukos G (2011). Chimeric 16S rRNA sequence formation and detection in Sanger and 454-pyrosequenced PCR amplicons. Genome Res.

[109] Edgar RC (2013). UPARSE: highly accurate OTU sequences from microbial amplicon reads. Nat Methods.

[110] Quast C, Pruesse E, Yilmaz P, Gerken J, Schweer T, Yarza P (2013). The SILVA ribosomal RNA gene database project: improved data processing and web-based tools. Nucleic Acids Res..

[111] Friedman J, Alm EJ (2012). Inferring correlation networks from genomic survey data. PLoS Comput Biol.

