# Supplementary material for: Core gut microbes Cloacibacterium and Aeromonas associated with different gastropod species could be persistently transmitted across multiple generations
Source: Microbiome. 2023 Nov 29;11:267. doi: 10.1186/s40168-023-01700-0 (PMC10685545; doi:10.1186/s40168-023-01700-0)
Supplement: Supplementary file 3 — Additional file 2: Fig. S1. Overview of the gut microbiome of freshwater gastropods at the genus (Top 50) and phylum level. The yellow and red columns indicated the relative abundance of gut microbes of wild and cultured populations, respectively. Cultured snails refer to the laboratory snails cultured under laboratory conditions. Fig. S2. Overview of the gut microbiome of freshwater gastropods at the genus and phylum level. a-f Gut bacterial genera of cultured freshwater gastropods, including B. straminea (n =6), B. aeruginosa (n =8), P. acuta (n =4), P. corneus (n =6), B. glabrata (n =7) and B. pfeif eri (n =8). g-k Gut bacterial genera of wild-caught freshwater snails, including P. canaliculata (n =3), B. straminea (n =14), B. aeruginosa (n =6), P. acuta (n =6) and P. corneus (n =6). l The relative abundance of bacterial phyla. Cultured snails refer to the laboratory snails cultured under laboratory conditions. Fig. 3. Difference in gut microbiota between wild and cultured freshwater gastropods. a At the genus level. b A linear discriminant analysis (LDA) effect size (LEfSe) analysis. Cultured snails refer to the laboratory snails cultured under laboratory conditions. Fig. S4. The relative abundance of gut microbiota of Wild and Wildt gastropods. Wild: wild-caught snails. WildT: wild-caught snails being transferred to the laboratory. Fig. S5. Overview of vertical shifts of gut microbes in Planorbarius corneus gastropods over generations. a PCoA of gut microbiota communities of wild and the F1 generation of Planorbarius corneus snails. b PCoA. c The number of OTUs are shared or unique to sample types. PlCmat: Maternal P. corneus snails. PlCoffsp: the offspring (F1 genernation) of maternal P. corneus snails reared under laboratory conditions. Fig. S6. Longitudinal shifts (from 0 days to 450 days) in the gut microbiome community diversity of freshwater snails. a Shannon index. b Observed species in the gut microbiome community diversity of freshwater snails. a Shann [file 40168_2023_1700_MOESM2_ESM.pdf]

Supplementary information for

Core gut microbes *Cloacibacterium* and *Aeromonas* associated with different gastropod species could be persistently transmitted across multiple generations

Datao Lin[1,2*], Jinni Hong[3], Benjamin Sanogo[1,4], Shuling Du[1,2], Suoyu Xiang[1,2], Jerome Ho-Lam Hui[5], Tao Ding[1,2*], Zhongdao Wu[1,2*] and Xi Sun[1,2*]

[1]Department of Parasitology, Key Laboratory of Tropical Disease Control (Ministry of Education), Zhongshan School of Medicine, Sun Yat-Sen University, Guangzhou, China. [2]Provincial Engineering Technology Research Center for Diseases-vectors Control and Chinese Atomic Energy Agency Center of Excellence on Nuclear Technology Applications for Insect Control, Sun Yat-Sen University, Guangzhou, China. [3]Department of Traditional Chinese Medicine, Guangdong Provincial People's Hospital, Guangdong Academy of Medical Sciences, Southern Medical University, Guangzhou, China. [4]Laboratory of Parasitology, Institut National de Recherche en Sante Publique, Bamako, Mali. [5]State Key Laboratory of Agrobiotechnology, School of Life Science, The Chinese University of Hong Kong, Hong Kong, China.

[*]**Correspondence**:
sunxi2@mail.sysu.edu.cn, wuzhd@mail.sysu.edu.cn, dingt8@mail.sysu.edu.cn, lindt5@mail.sysu.edu.cn

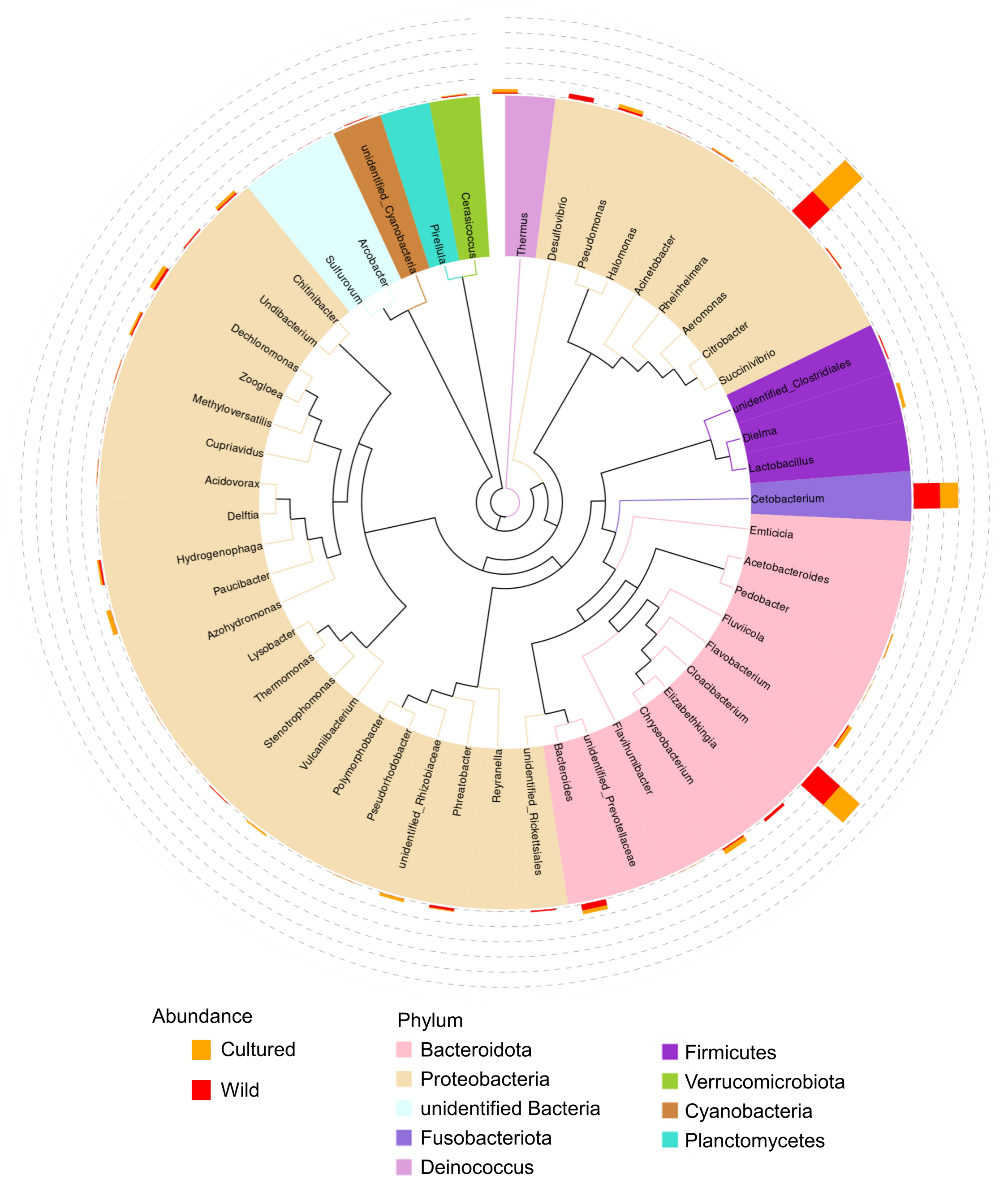

Fig. S1 Overview of the gut microbiome of freshwater gastropods at the genus (Top 50) and phylum level. The yellow and red columns indicated the relative abundance of gut microbes of wild and cultured populations, respectively. Cultured snails refer to the laboratory snails cultured under laboratory conditions.

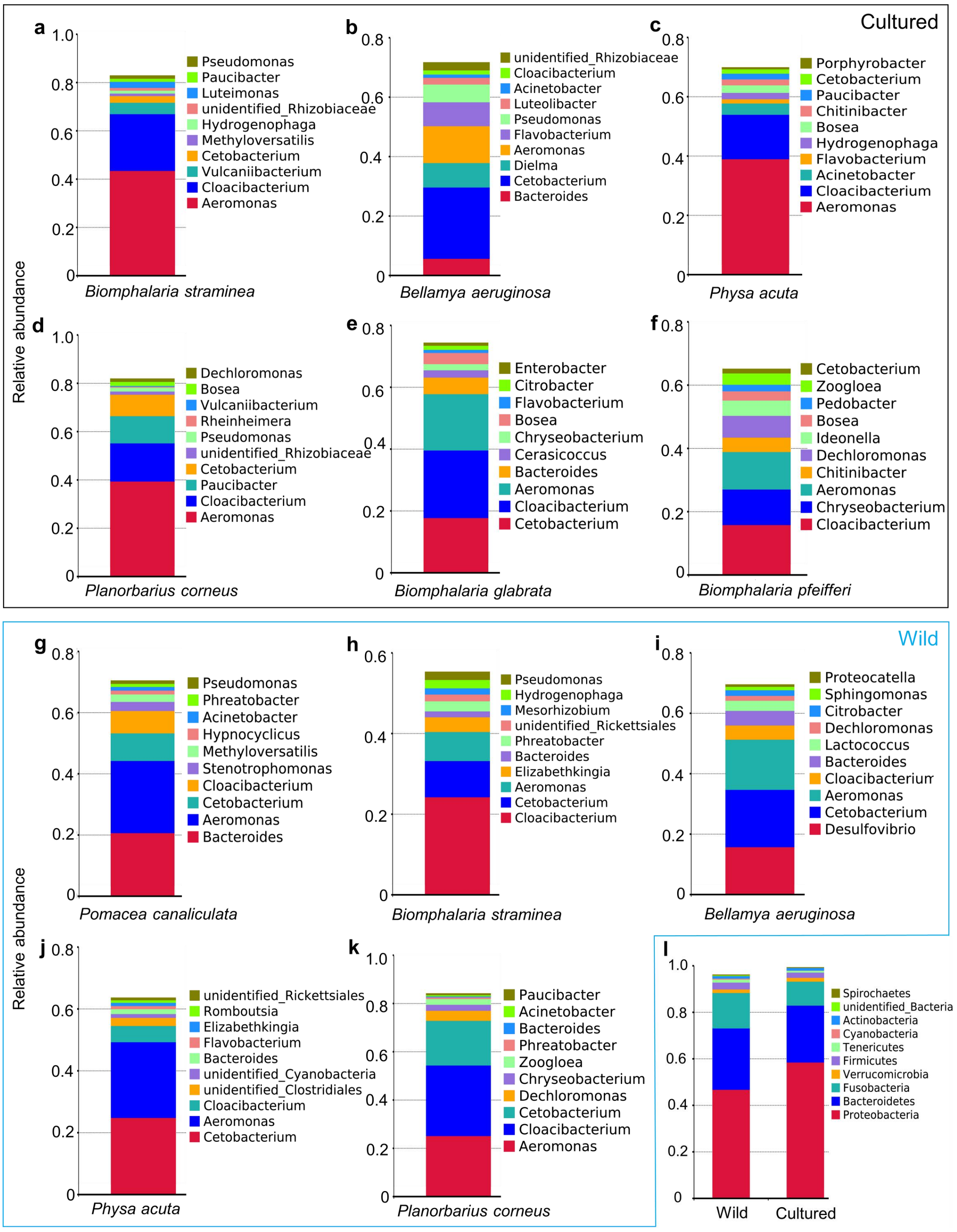

Fig. S2 Overview of the gut microbiome of freshwater gastropods at the genus and phylum level. **a-f** Gut bacterial genera of cultured freshwater gastropods, including *B. straminea* (n =6), *B. aeruginosa* (n =8), *P. acuta* (n =4), *P. corneus* (n =6), *B. glabrata* (n =7) and *B. pfeifferi* (n =8). **g-k** Gut bacterial genera of wild-caught freshwater snails, including *P. canaliculata* (n =3), *B. straminea* (n =14), *B. aeruginosa* (n =6), *P. acuta* (n =6) and *P. corneus* (n =6). **l** The relative abundance of bacterial phyla. Cultured snails refer to the laboratory snails cultured under laboratory conditions.

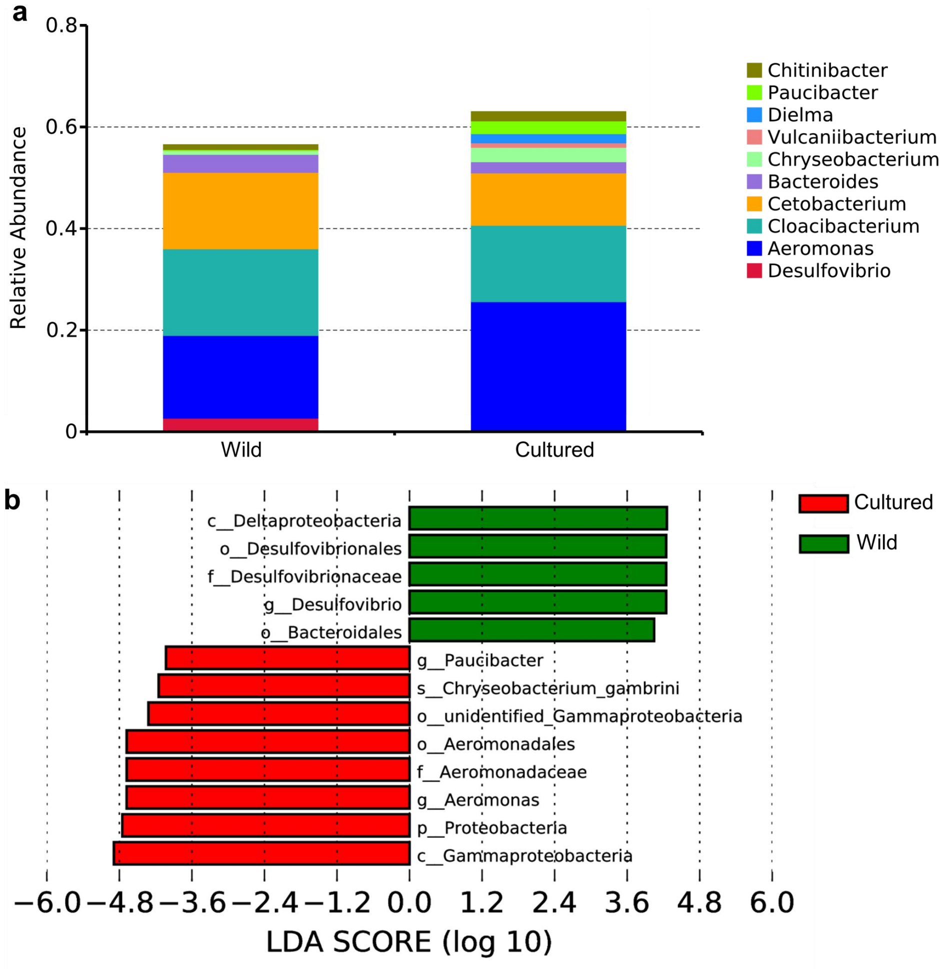

Fig. 3 Difference in gut microbiota between wild and cultured freshwater gastropods. **a** At the genus level. **b** A linear discriminant analysis (LDA) effect size (LEfSe) analysis. Cultured snails refer to the laboratory snails cultured under laboratory conditions.

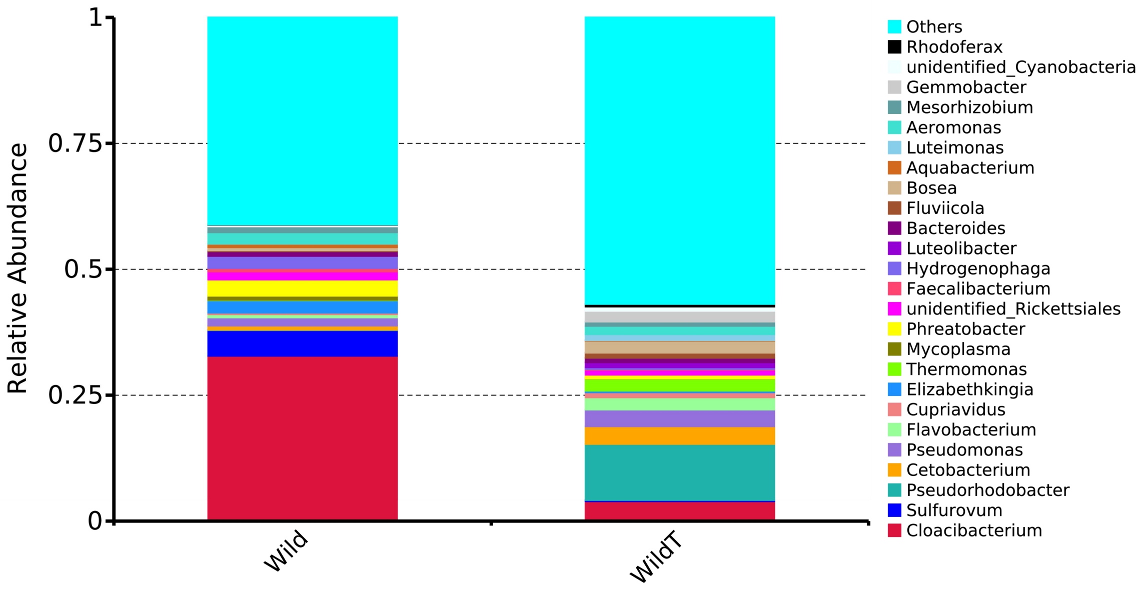

Fig. S4 The relative abundance of gut microbiota of Wild and Wildt gastropods. Wild: wild-caught snails. WildT: wild-caught snails being transferred to the laboratory.

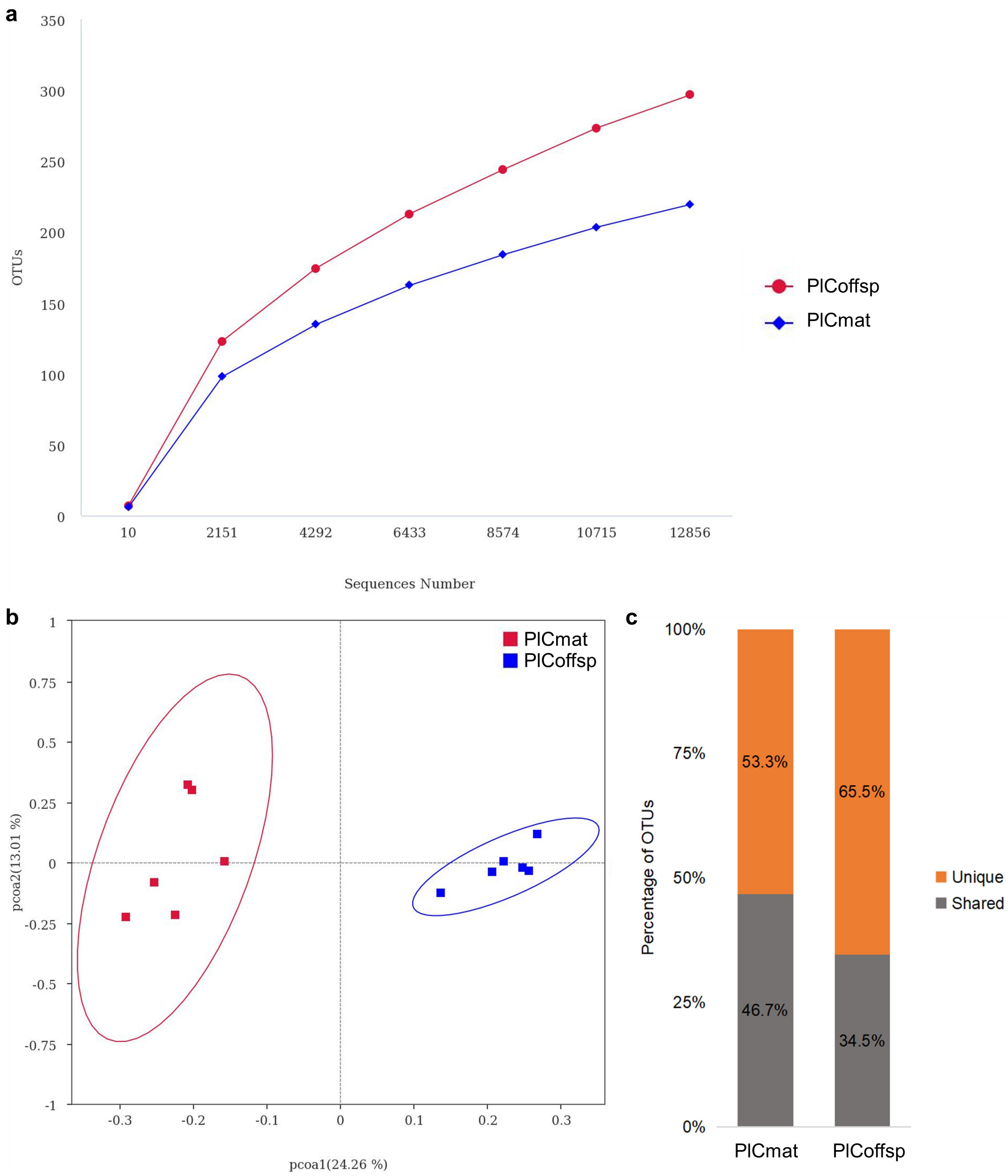

Fig. S5 Overview of vertical shifts of gut microbes in *Planorbarius corneus* gastropods over generations. **a** PCoA of gut microbiota communities of wild and the F1 generation of *Planorbarius corneus* snails. **b** PCoA. **c** The number of OTUs are shared or unique to sample types. PlCmat: Maternal *P. corneus* snails. PlCoffsp: the offspring (F1 genernation) of maternal *P. corneus* snails reared under laboratory conditions.

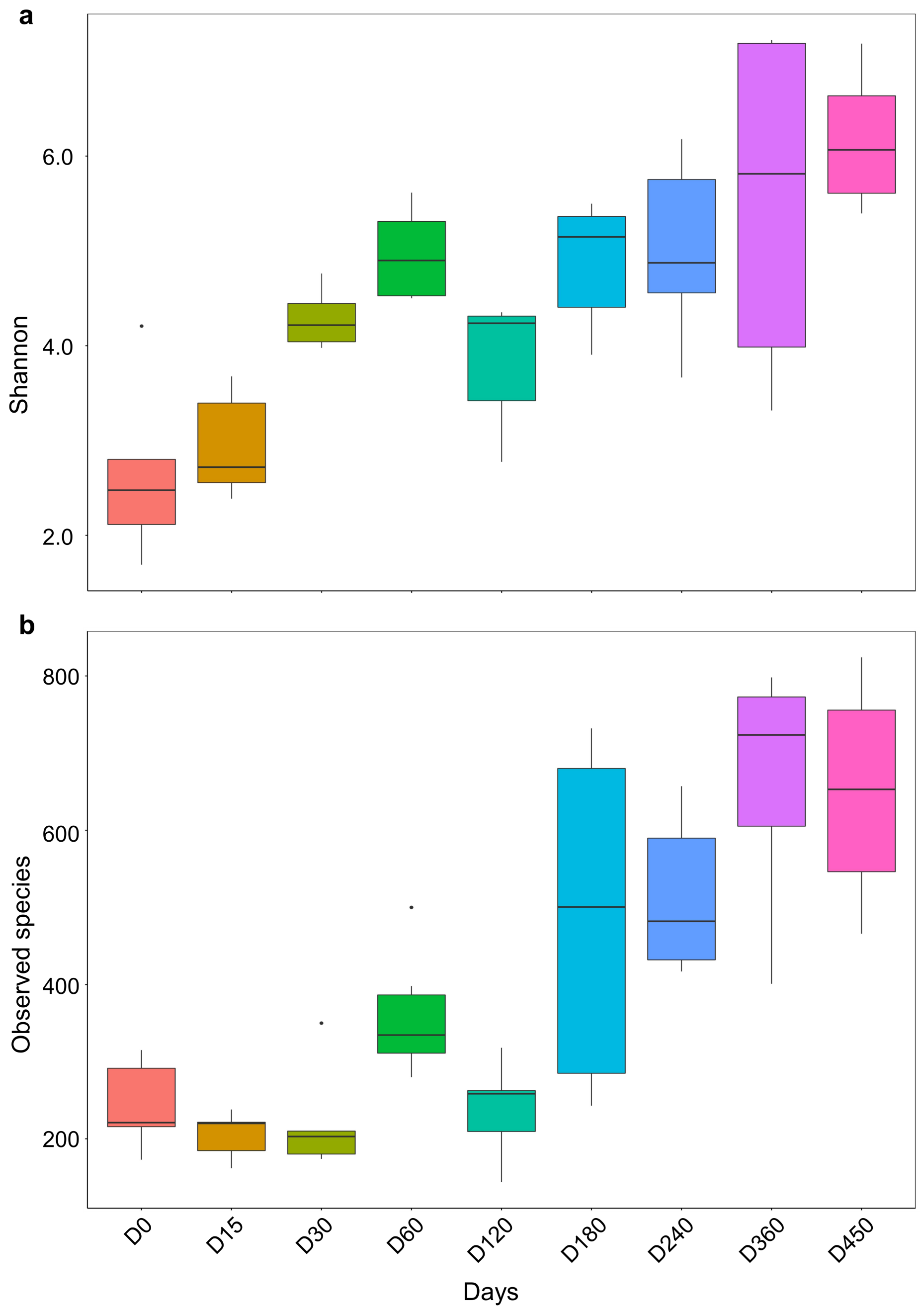

Fig. S6 Longitudinal shifts (from 0 days to 450 days) in the gut microbiome community diversity of freshwater snails. **a** Shannon index. **b** Observed species index.

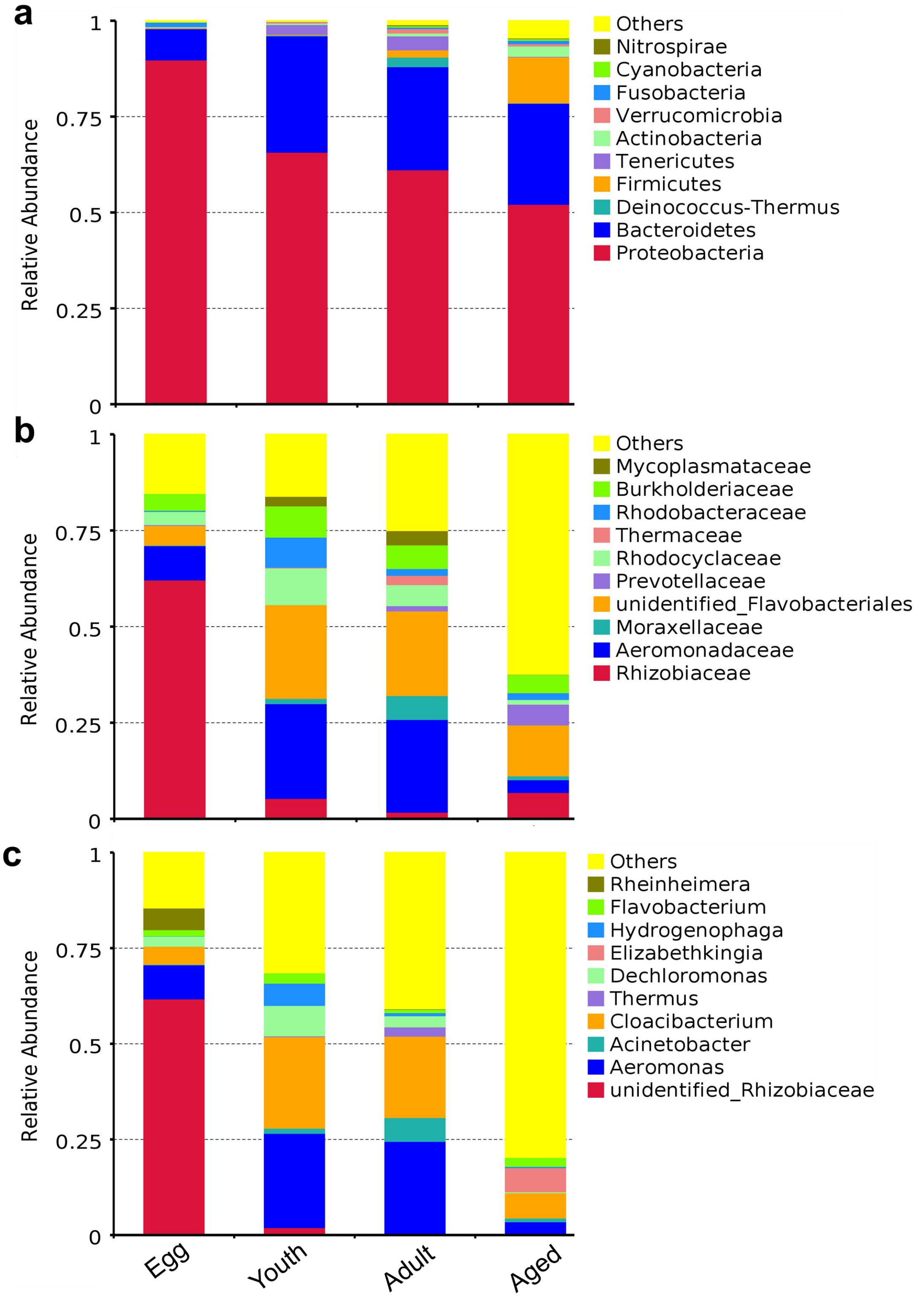

Fig. S7 The relative abundance of gut microbiota of freshwater gastropods across developmental stages. **a** At the phylum level. **b** At the family level. **c** At the genus level.

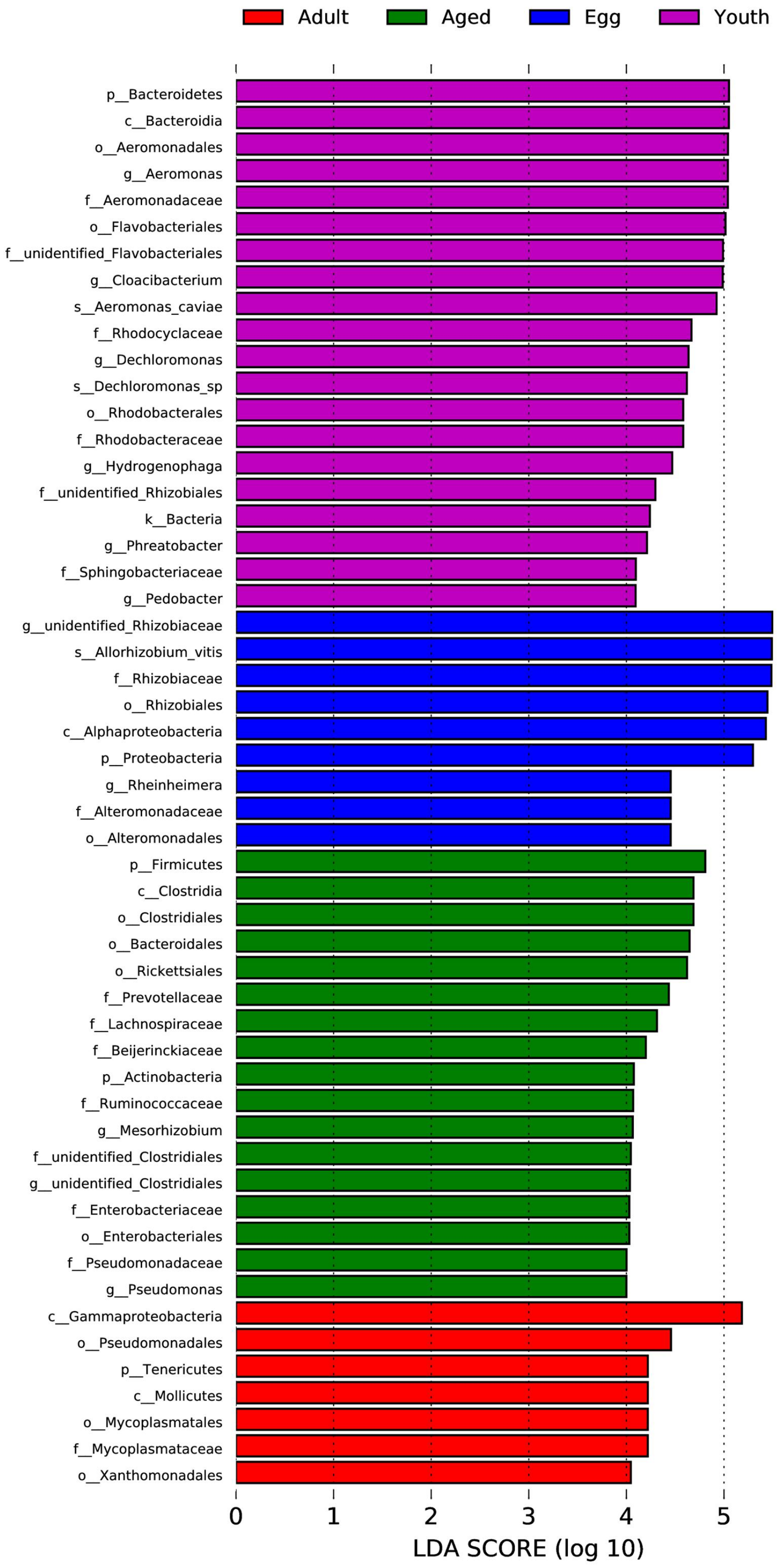

Fig. S8 Differential gut bacterial taxa analyzed by LEfSe analysis with LDA score >4 across developmental stages.

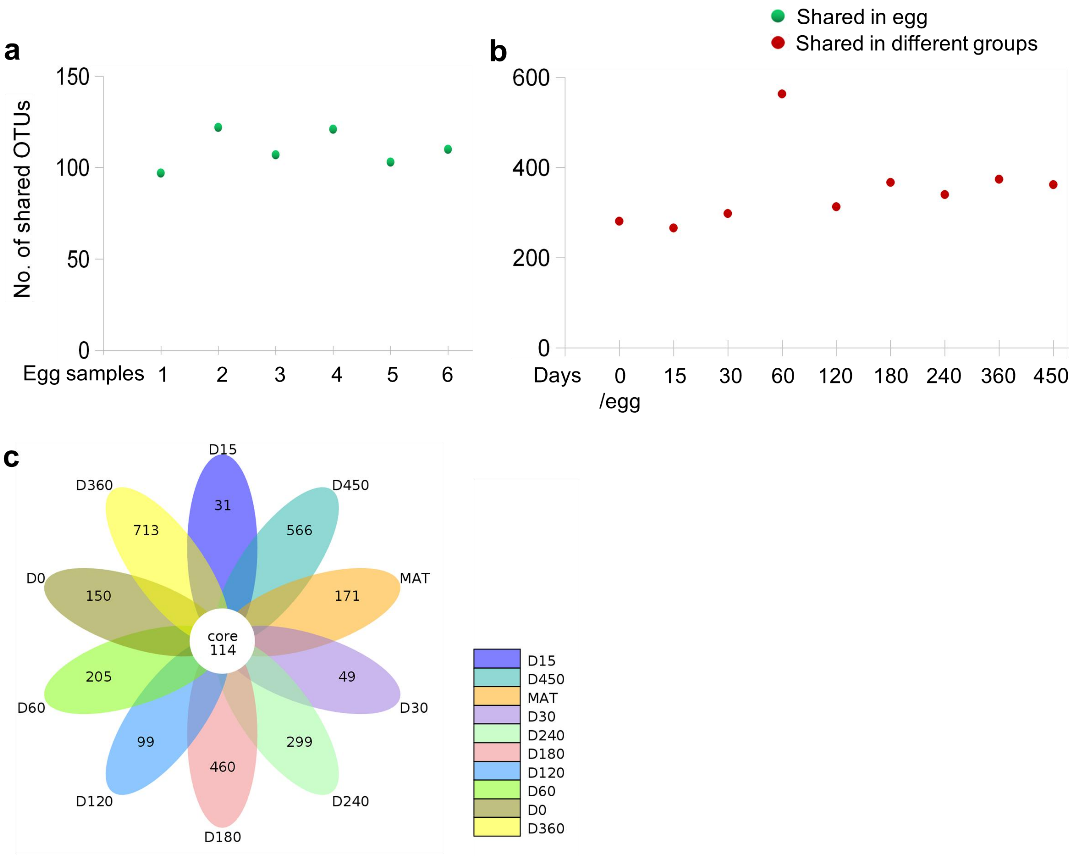

Fig. S9 a Percentage of OTUs from maternal snails transmitted to eggs. **b** Percentage of OTUs from maternal snails transmitted to snails at different developmental stages. **c** Venn diagram of the "core" microbes shared among different developmental stages.

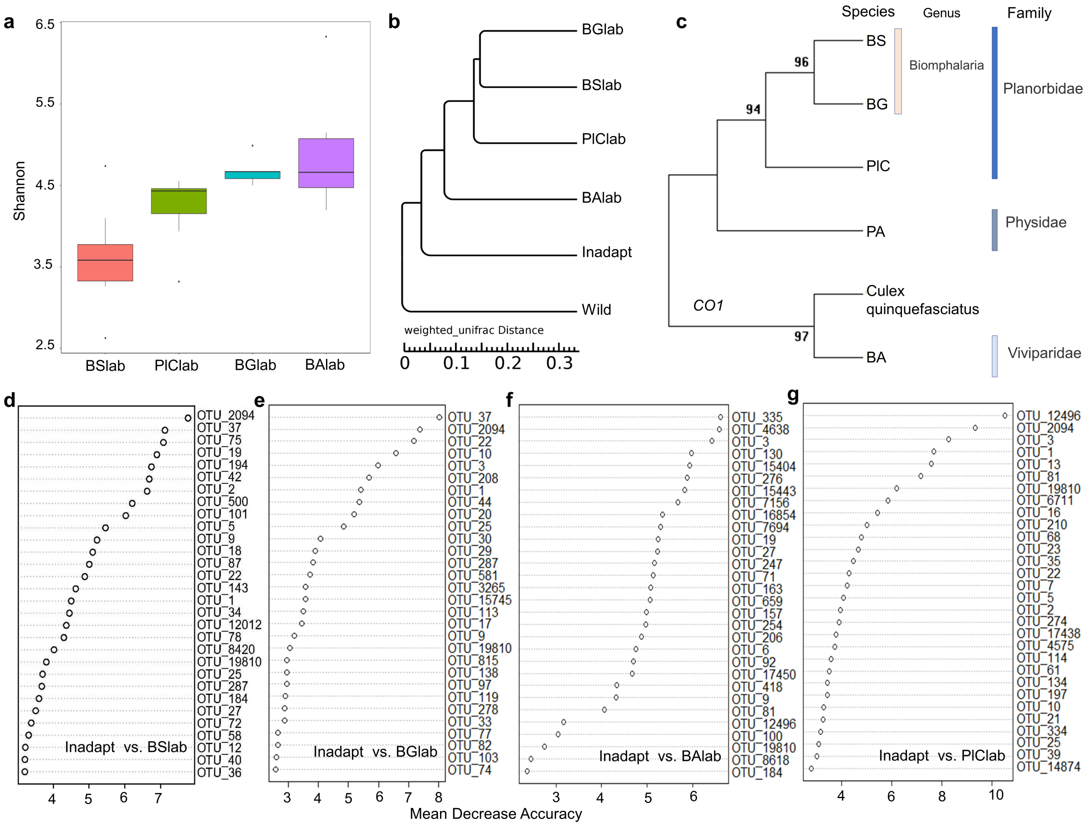

Fig. S10 Adaptation of gastropods is associated with gut microbiota. **a** Shannon index. **b** UPGMA analysis conducted based on binary Jaccard distance. **c** Phylogenetic evolutionary constructed based on the maximum-likelihood algorithm for *CO1* gastropod sequences. **d-g** Top 30 fitness-related OTUs compared with different local populations are shown.

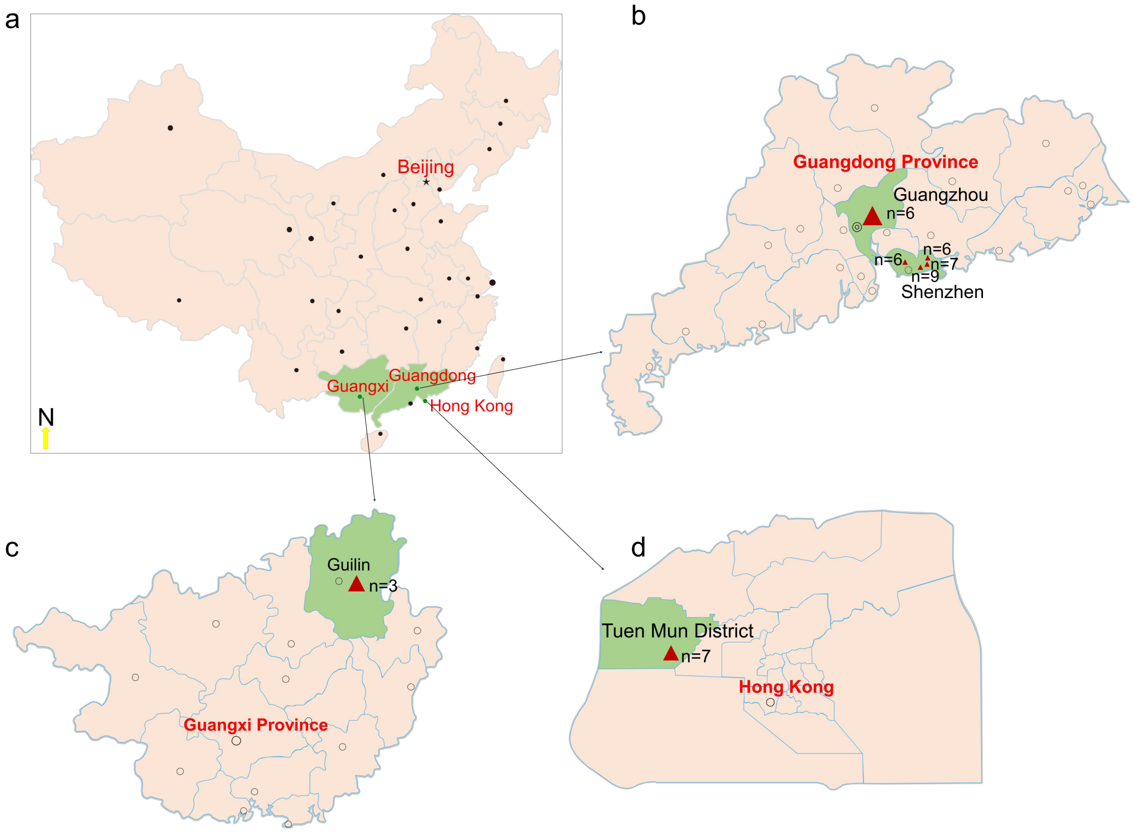

Fig. S11 Overview of sampling study of wild snails. **a** Wild snails were collected from Guangdong (**b**), Guangxi (**c**), and Hong Kong (**d**) in China. The major sites were marked on the maps. The red triangles indicate the sampling sites.

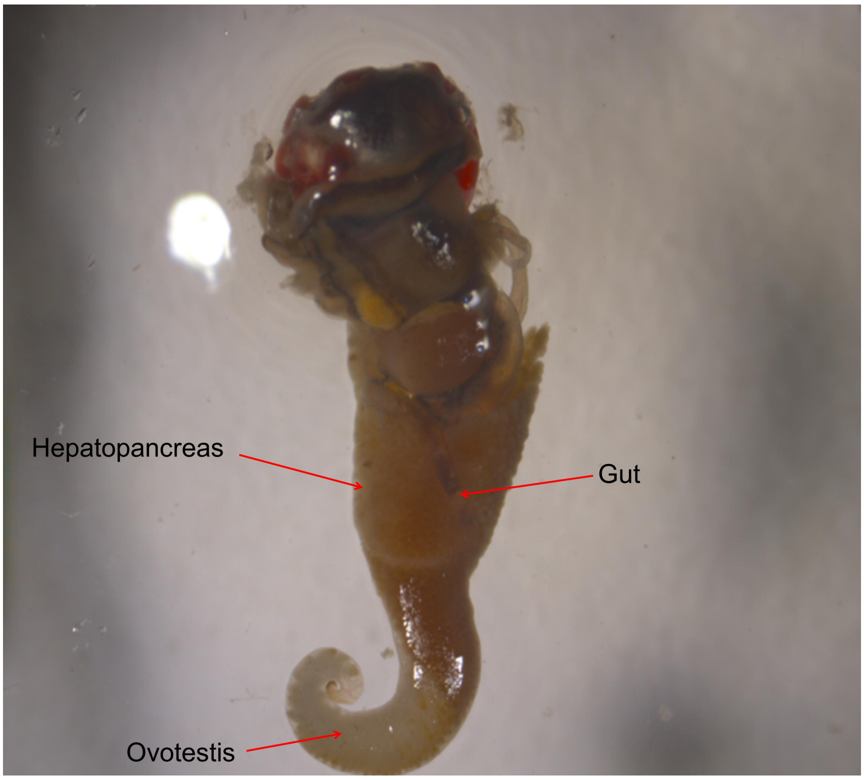

Fig. S12 Picture of body tissue of *Biomphalaria straminea*.
